# Polyadenylation-Dependent Control of Long Noncoding RNA Expression by the Poly(A)-Binding Protein Nuclear 1

**DOI:** 10.1371/journal.pgen.1003078

**Published:** 2012-11-15

**Authors:** Yves B. Beaulieu, Claudia L. Kleinman, Anne-Marie Landry-Voyer, Jacek Majewski, François Bachand

**Affiliations:** 1 RNA Group, Department of Biochemistry, Université de Sherbrooke, Sherbrooke, Québec, Canada; 2 Department of Human Genetics, McGill University, Montreal, Québec, Canada; Massachusetts General Hospital, Howard Hughes Medical Institute, United States of America

## Abstract

The poly(A)-binding protein nuclear 1 (PABPN1) is a ubiquitously expressed protein that is thought to function during mRNA poly(A) tail synthesis in the nucleus. Despite the predicted role of PABPN1 in mRNA polyadenylation, little is known about the impact of PABPN1 deficiency on human gene expression. Specifically, it remains unclear whether PABPN1 is required for general mRNA expression or for the regulation of specific transcripts. Using RNA sequencing (RNA–seq), we show here that the large majority of protein-coding genes express normal levels of mRNA in PABPN1–deficient cells, arguing that PABPN1 may not be required for the bulk of mRNA expression. Unexpectedly, and contrary to the view that PABPN1 functions exclusively at protein-coding genes, we identified a class of PABPN1–sensitive long noncoding RNAs (lncRNAs), the majority of which accumulated in conditions of PABPN1 deficiency. Using the spliced transcript produced from a snoRNA host gene as a model lncRNA, we show that PABPN1 promotes lncRNA turnover via a polyadenylation-dependent mechanism. PABPN1–sensitive lncRNAs are targeted by the exosome and the RNA helicase MTR4/SKIV2L2; yet, the polyadenylation activity of TRF4-2, a putative human TRAMP subunit, appears to be dispensable for PABPN1–dependent regulation. In addition to identifying a novel function for PABPN1 in lncRNA turnover, our results provide new insights into the post-transcriptional regulation of human lncRNAs.

## Author Summary

In eukaryotic cells, protein-coding genes are transcribed to produce pre-messenger RNAs (pre–mRNAs) that are processed at the 3′ end by the addition of a sequence of poly-adenosine. This 3′ end poly(A) tail normally confers positive roles to the mRNA life cycle by stimulating nuclear export and translation. The fundamental role of mRNA polyadenylation is generally mediated by the activity of poly(A)-binding proteins (PABPs) that bind to the 3′ poly(A) tail of eukaryotic mRNAs. In the nucleus, the evolutionarily conserved poly(A)-binding protein PABPN1 is thought to be important for gene expression, as it stimulates mRNA polyadenylation in biochemical assays. Using a high-throughput sequencing approach that quantitatively measures the level of RNA expressed from all genes, we addressed the global impact of a PABPN1 deficiency on human gene expression. Notably, we found that most mRNAs were normally expressed in PABPN1–deficient cells, a result inconsistent with a role for PABPN1 in general mRNA metabolism. Surprisingly, our genome-wide analysis unveiled a new function for PABPN1 in a polyadenylation-dependent pathway of RNA decay that targets non-protein coding genes. Our discovery that PABPN1 functions in the regulation of noncoding RNAs raises the possibility that oculopharyngeal muscular dystrophy, a disease associated with mutations in the *PABPN1* gene, is caused by defective expression of noncoding RNAs.

## Introduction

Poly(A)-binding proteins (PABPs) play essential roles in eukaryotic gene expression. Normally, PABPs bind the poly(A) tract of mRNAs to confer positive roles in the mRNA life cycle, such as stability and translational activity. Two evolutionarily conserved, yet structurally different PABPs bind the poly(A) tract of mRNAs in most eukaryotic cells: PABPC1 in the cytoplasm and PABPN1/PABP2 in the nucleus. The product of the *PABPN1* gene was originally identified as a factor that stimulates the synthesis of RNA poly(A) tails *in vitro* [1]. At the biochemical level, the affinity of PABPN1 for synthetic poly(A) tails is in the nanomolar range and requires the single RNA recognition motif and a C-terminal arginine-rich domain for optimal and specific interaction with poly(A) [2]. Experiments using *in vitro* polyadenylation assays have led to a model in which PABPN1 stimulates processive poly(A) synthesis by direct and simultaneous interactions with the growing mRNA poly(A) tail and the poly(A) polymerase [3], [4], defining PABPN1 as a general mRNA polyadenylation factor [1], [3], [5]. Consistent with a role in polyadenylation, siRNA-mediated gene silencing experiments in primary mouse myoblasts showed global shortening of mRNA poly(A) tails after PABPN1 depletion [6]. In *Drosophila*, specific *pabp2* mutants also result in shorter poly(A) tails [7]. For these latter studies, however, the effect of the reported poly(A) tail shortening on global mRNA expression was not addressed. In fission yeast, a genome-wide analysis of gene expression changes in cells deleted for *pab2*, which encodes the PABPN1 ortholog, revealed that the expression of most protein-coding genes was unaffected, indicating that Pab2 does not function as a general mRNA maturation factor in this organism [8]. Similarly, PABPN1 appears dispensable for general mRNA expression in *C. elegans*, as this protein can be depleted from larvae without significant effects on mRNA levels and global translation [9]. As the consequences of PABPN1 deficiency on global gene expression have not been examined in human cells, the requirement of PABPN1 for general mRNA synthesis remains elusive.

A detailed understanding of the role of PABPN1 in gene expression is significant, as the human genetic disorder oculopharyngeal muscular dystrophy (OPMD) is linked to mutations in the *PABPN1* gene. OPMD usually appears between the fourth and sixth decade of life and is primarily associated with drooping eyelids, swallowing difficulties, and proximal limb weakness [10]. Although OPMD is a relatively rare disease, cases have now been reported in more than 35 countries [10] with greater prevalence in the French Canadian [11], Bukharian Jew [12], and New Mexican Hispanic [13] populations. OPMD is caused by short expansions of GCG-repeats in the first exon of the *PABPN1* gene, which produce proteins with a stretch of 12–17 alanines [14], whereas the amino-terminal region of PABPN1 normally contains a stretch of 10 alanines. The underlying mechanism by which *PABPN1* mutations cause OPMD is still unclear.

Because OPMD is mainly restricted to ocular and pharyngeal muscles, it has been difficult to reconcile how defects in a cellular process as fundamental as mRNA polyadenylation could result in such a tissue-specific disease. In addition, Calado *et al.* showed that poly(A) tail length is not affected in myoblasts from individuals with OPMD [15], suggesting that the molecular basis of OPMD may result from a deficiency in a previously uncharacterized role of PABPN1. Consistent with this view, studies in yeast and humans have recently identified functions for this poly(A)-binding protein in gene-specific regulation. A screen to isolate modulators of mRNA cleavage site decision has recently identified PABPN1 as a regulator of this process [16]. Specifically, PABPN1 was shown to control the use of alternative polyadenylation sites for a specific set of human protein-coding genes, most likely by competing with the 3′ end processing machinery at weak polyadenylation signals. In fission yeast, Pab2 promotes the decay of specific meiotic transcripts during the fission yeast mitotic cycle via physical interactions with the Mmi1 protein, which recognizes *cis* elements within meiotic mRNAs [17], [18], [19]. Pab2 also functions in a nuclear pre-mRNA decay pathway that controls the expression of specific intron-containing genes [20]. In both of these cases, Pab2 cooperates with the exosome complex, an evolutionarily conserved RNA degradation machinery that contains 3′→5′ exonuclease and endonuclease activities 21,22. Whether human PABPN1 is involved in a polyadenyation-dependent pathway of RNA decay is currently unknown.

To investigate the global effect of a PABPN1 deficiency on human gene expression, we performed a transcriptome-wide analysis of PABPN1–depleted cells using RNA sequencing. We find that the expression of most protein-coding genes is not affected by the loss of PABPN1, with less than 5% of the expressed genes showing misregulation at the mRNA level. Unexpectedly, a deficiency of PABPN1 resulted in the accumulation of a significant number of long noncoding RNAs (lncRNAs). By studying how PABPN1 promotes the turnover of the lncRNA encoded from the SNORD60-Host Gene (*SHG60*), we found a mechanism of RNA decay that requires lncRNA polyadenylation and the RNA exosome complex. Our findings thus define a novel function for PABPN1 in the regulation of human lncRNAs.

## Results

### Normal mRNA synthesis in PABPN1–depleted human cells

**Figure 1:**
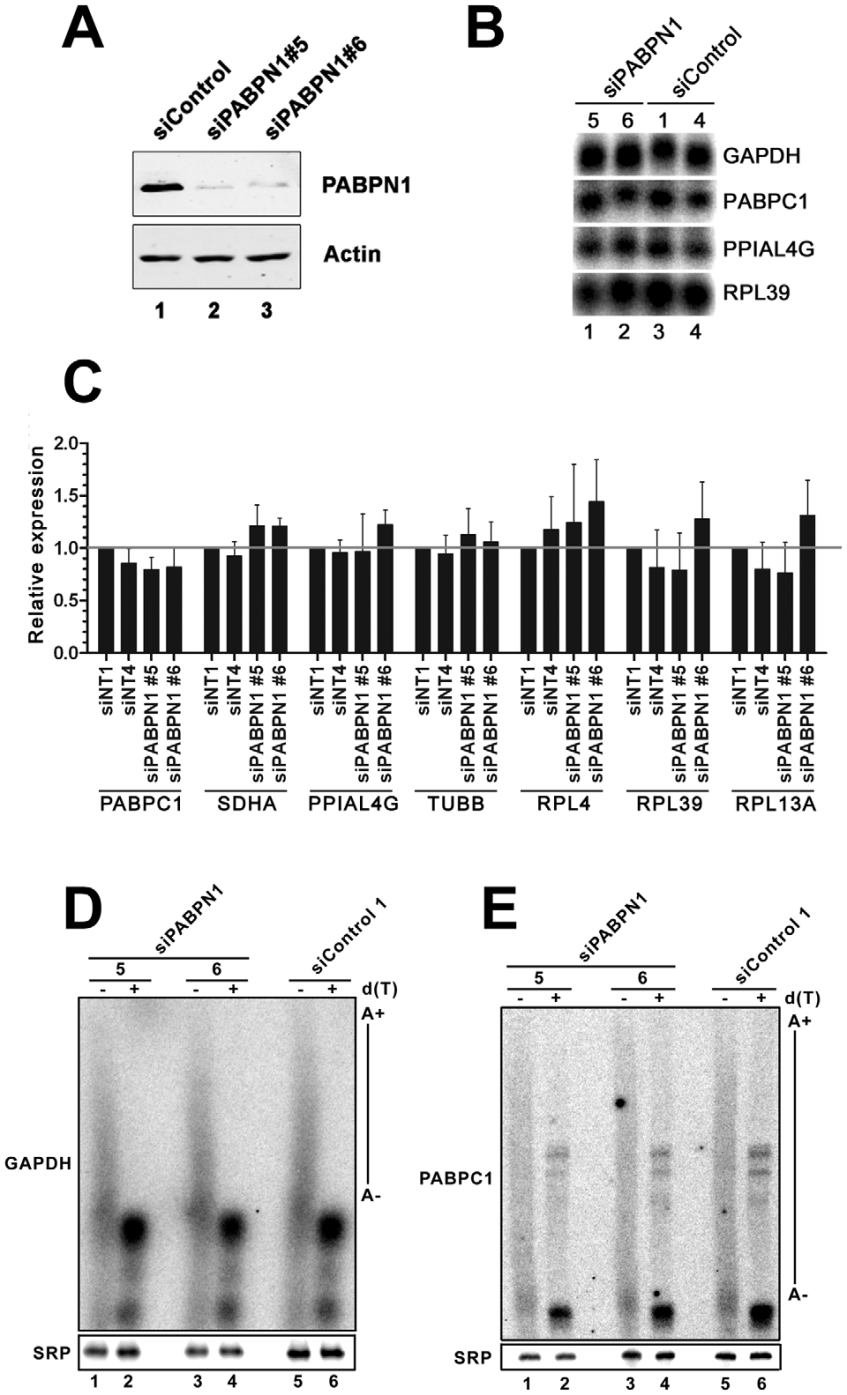
Expression and polyadenylation of housekeeping genes in PABPN1–depleted cells. (A) Western blot analysis of total extracts prepared from HeLa cells treated with PABPN1–specific (lanes 2–3) and control (lane 1) siRNAs for 72 hrs. (B) Northern blot analysis of total RNA prepared from HeLa cells treated with PABPN1–specific siRNAs (siRNA #5 and #6: lanes 1–2) and control siRNA (siRNA #1 and #4: lanes 3–4) for 72 hrs. The blot was probed for the GAPDH, PABPC1, peptidylprolyl isomerase A-like 4A (PPIAL4G), and ribosomal protein L39 (RPL39) mRNAs. (C) The expression levels of seven human genes were analyzed using real-time quantitative PCR in cells treated with control (siNT1 and siNT4) and PABPN1–specific (si#5 and si#6) siRNAs. The expression levels are relative to siNT1-treated cells and normalized to the GAPDH mRNA. The data and error bars represent the average and standard deviation from three independent experiments. (D–E) Total RNA prepared from HEK293T cells treated with PABPN1–specific siRNAs (siRNA#5: lanes 1–2; siRNA#6: lanes 3–4) and control siRNA#1 (lanes 5–6) siRNAs was treated with RNase H in the presence of DNA oligonucleotides complementary to 3′UTR sequences of the GAPDH (D) and PABPC1 (E) mRNAs. RNase H reactions were performed in the presence (+) and absence (−) of oligo d(T). The signal recognition particle (SRP) RNA was used as a loading control.

We previously reported that the abundance and polyadenylation status of most mRNAs were not affected in fission yeast cells deleted for the gene encoding the human PABPN1 homolog [8]. In contrast, the requirement for PABPN1 in mRNA expression has not been clearly addressed in humans. We therefore depleted PABPN1 from human cell lines using independent PABPN1–specific siRNAs, routinely depleting over 90% of the total PABPN1 protein (Figure 1A, lanes 2–3). Remarkably, no noticeable changes were detected in the levels of several transcripts as determined by northern blotting (Figure 1B) and qRT-PCR (Figure 1C). Similar results were obtained using RNA prepared from HEK293 and U2OS cells (data not shown). We also analyzed the 3′ end of specific mRNAs by treating total RNA prepared from PABPN1–depleted and control cells with RNase H. RNase H treatment in the presence of a DNA oligonucleotide complementary to a region located in the 3′ UTR of an mRNA will release heterogeneous 3′ fragments, as a consequence of different poly(A) tail lengths. The addition of oligo d(T) to the RNase H reaction causes this heterogeneous population of 3′ fragments to collapse into discrete products, indicating the position of polyadenylation sites. As can be seen for the *GAPDH* and *PABPC1* mRNAs, poly(A) tail lengths and cleavage site decisions were similar between PABPN1–depleted and control cells (Figure 1D–1E, compare lanes 1–4 to 5–6). From these results, we conclude that the expression and polyadenylation of several mRNAs occur normally in cells deficient for PABPN1.

### Long noncoding (lnc) RNA expression is more affected than mRNA expression by a deficiency in PABPN1

**Figure 2:**
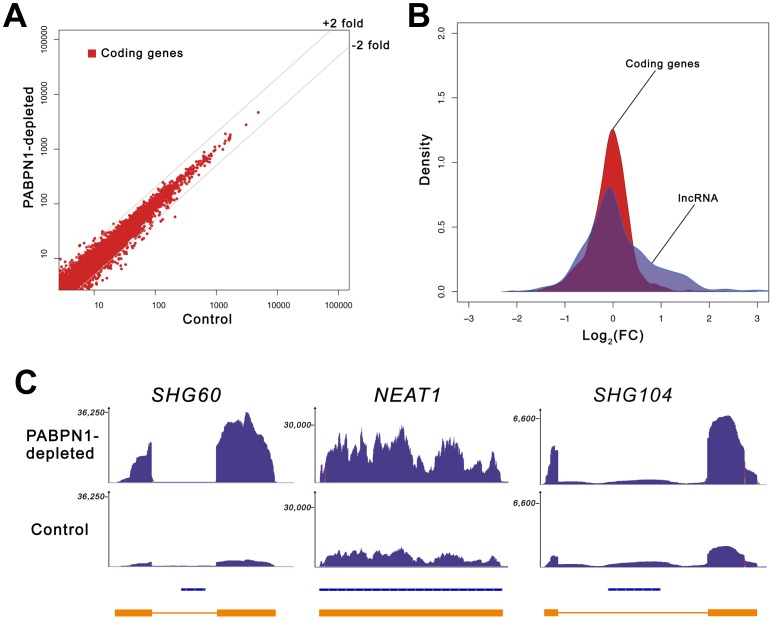
Loss of PABPN1 induces specific changes in gene expression. (A) Comparison of gene expression changes in PABPN1–depleted (*y*-axis) and control (*x*-axis) cells, measured in reads per kilobase per million reads (RPKM). Coding genes (red dots) are defined as all UCSC genes with an associated protein ID. (B) Shown is the distribution of log ratios/fold-change (FC) in expression levels for protein-coding (red) and lncRNA (blue) genes using Kernel density estimation. The original histograms are shown in Figure S1. The set of lncRNA genes comprises all genes from the lincRNA catalog [26], lncRNA database [82], and genes from the noncode database [83] longer than 200-nt. (C) RNA-seq read distribution along three lncRNA genes for PABPN1–depleted (top row) and control (middle row) cells. Bottom row shows gene annotations for the corresponding regions: Refseq genes (blue) and inferred gene structure from RNA-seq data (orange). *SHG60* and *SHG104* (SNORD60 and SNORD104 Host Genes, respectively); *NEAT1* (nuclear enriched abundant transcript 1).

The lack of change in the expression level of selected mRNAs prompted us to measure the global impact of PABPN1 deficiency on the human transcriptome. We therefore conducted an expression profiling experiment using next-generation sequencing (RNA-Seq) and obtained >190 millions of 100-bp paired-end reads for both PABPN1–depleted and control HeLa cells. Sequence reads were mapped to the human genome with a spliced read aligner [23], and gene expression was computed based on the number of exonic reads mapping to each gene, normalized by transcript length and total number of reads (RPKM) [24]. A global comparison of the RNA-seq data between PABPN1–depleted and control cells is presented in Figure 2A. Notably, expression levels of most protein-coding genes were unaffected in PABPN1–depleted cells, with only ∼3% showing more than a 2-fold change: 78 and 227 genes demonstrated increased and decreased mRNA levels, respectively, out of 11,572 coding genes with RPKM >1 (Table S1). This distribution is asymmetrical (Figure 2B), with more protein-coding genes showing decreased expression due to a deficiency in PABPN1 (skewness 0.33, D'Agostino K-squared test *p*-value <2.2e-16). We also measured the impact of PABPN1 deficiency on mRNAs exclusively (using a dataset that excluded loci for which protein-coding and noncoding RNA genes overlapped) and obtained similar results as with the more comprehensive coding gene dataset: ∼4% of the protein-coding genes showed changes greater than 2-fold (Table S2).

Because the loss of PABPN1 showed only minimal changes in mRNA expression, we investigated the impact of PABPN1 deficiency on the expression of noncoding (nc) RNAs. It should be noted that because the sequenced libraries were prepared using poly(A)^+^ transcripts, we do not expect to see reads mapping to known nonpolyadenylated ncRNAs (tRNAs, rRNAs, snoRNAs, and snRNAs) unless they are generated from a polyadenylated precursor. Notably, we found that a class of noncoding transcripts whose expression was substantially affected by PABPN1 deficiency was long noncoding (lnc) RNAs, defined as non-protein coding transcripts larger than 200 nucleotides [25]. From a conservative set of known/predicted 7693 human lncRNAs, we identified 469 lncRNAs that were expressed above background (RPKM >1 in at least one sample) in our cell line, which is consistent with results indicating that the expression of lncRNAs is highly tissue-specific [26]. Significantly, of the 469 lncRNAs detected in our cell line, 60 (13%) were upregulated by more than 2-fold in PABPN1–depleted cells, whereas 16 (3.4%) were down-regulated (Table S3). This distribution is different from the one obtained with protein-coding genes (*p*-value <1.5e-08, Student's t-test): it is assymetrical to the right in this case (Figure 2B), showing a clear excess of lncRNA genes with increased expression (skewness 0.87, D'Agostino K-squared test *p*-value <3.4e-06). The greater impact of a PABPN1 deficiency on lncRNA expression relative to mRNA expression is also observed by comparing the cumulative distribution of mRNAs and lncRNAs (Figure S2; *p*-value <2.2e-16, Kolmogorov-Smirnov test). Although most of the lncRNAs misregulated in PABPN1–depleted cells are still uncharacterized (Table S3), a few have been described previously, including NEAT1 [27], the RNase P RNA subunit RPPH1 [28], the BCYRN1/BC200 RNA [29], and the TUG1 RNA [30], all of which showed increased levels in PABPN1–depleted cells. Interestingly, some of the genes encoding PABPN1–sensitive lncRNAs are host to small nuclear (sno) RNAs and small Cajal body-specific (sca) RNAs in their introns (Figure 2C, see SHG60 and SHG104; Table S3). In humans, most snoRNAs reside in intronic sequences, a fraction of which are found in introns of non-protein coding genes [31]. Further analysis of the sequencing data indicated that the PABPN1–dependent upregulation of noncoding snoRNA-host transcripts was restricted to the spliced versions, as the overall level of intronic signal detected for the corresponding genes did not change between PABPN1–depleted and control cells (Figure 2C, see SHG60 and SHG104). Notably, the spliced RNA encoded from the SNORD60 Host Gene (SHG60) was the most upregulated lncRNA in PABPN1–depleted cells (Figure 2C; Table S3). 5′ and 3′ RACE experiments indicated that the intronic C/D box SNORD60 is processed from the intron of a 555-nt-long unspliced and polyadenylated precursor that generates a 355-nt-long spliced transcript with weak protein-coding potential (Figure S3). Consistent with the idea that the spliced transcript encoded from the SHG60 gene is noncoding, the two exons are poorly conserved between human and mouse; in contrast, the SNORD60 snoRNA is highly conserved.

**Figure 3:**
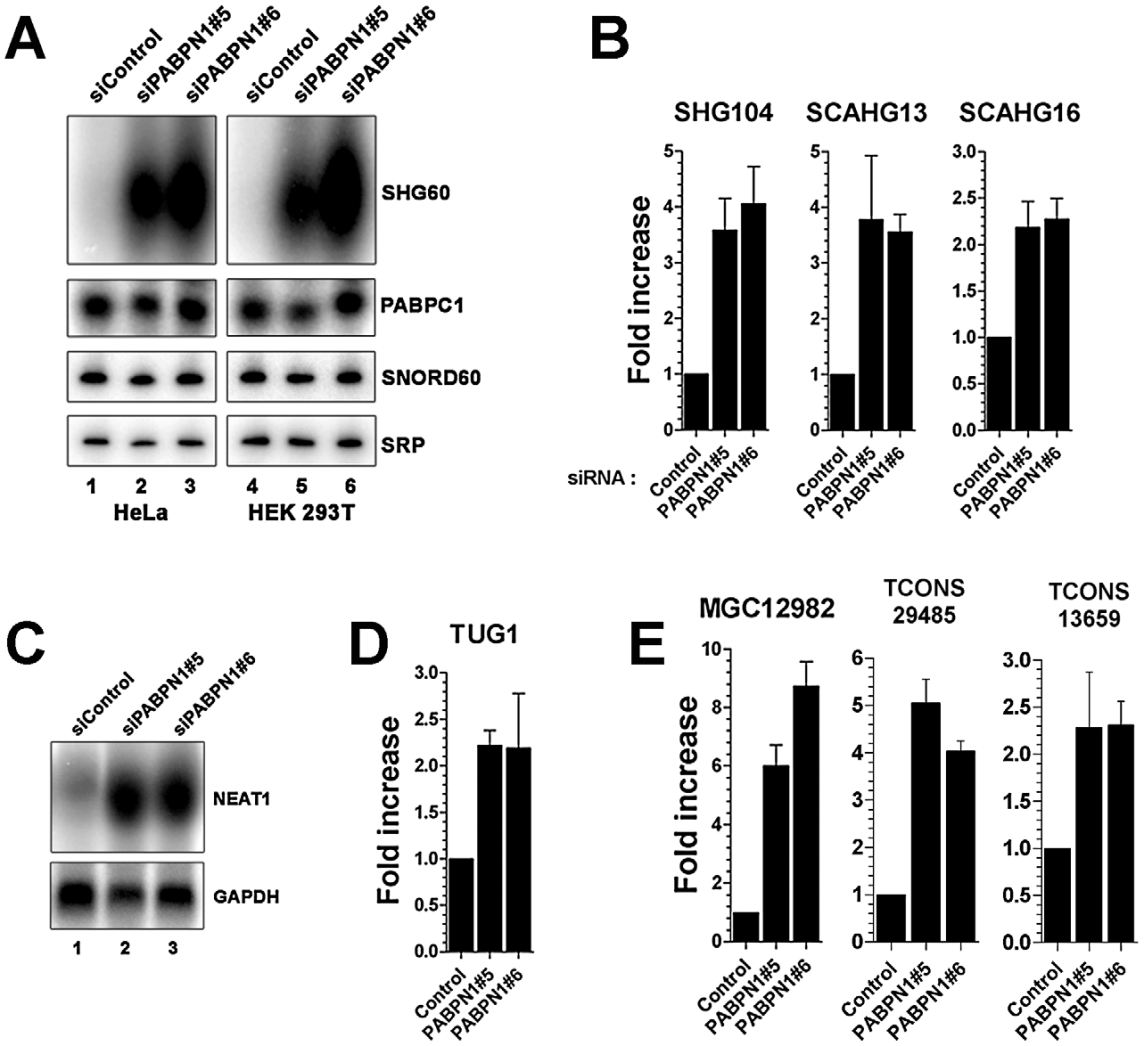
Accumulation of lncRNAs in PABPN1–depleted cells. (A) Northern blot analysis of total RNA prepared from HeLa (lanes 1–3) and HEK293T (lanes 4–6) cells treated with PABPN1–specific (lanes 2–3 and 5–6) and control (lanes 1 and 4) siRNAs. The blots were probed for the SHG60 lncRNA and the SNORD60 snoRNA. The PABPC1 mRNA and the signal recognition particle (SRP) RNA were used as loading controls. (B–E) PABPN1–sensitive lncRNAs identified by RNA-seq were confirmed by qRT-PCR (B, D, and E) and northern blot (C) by comparing RNA prepared from HeLa cells treated with control and PABPN1–specific siRNAs. Fold increases are relative to control siRNA and normalized to the GAPDH mRNA. The data and error bars represent the average and standard deviation from at least three independent experiments.

As the proportion of lncRNAs showing increased expression (13%) in conditions of PABPN1 deficiency was significantly greater than for downregulated lncRNAs (3.4%; *p*-value <3.4e-06, Chi-squared test) and protein-coding genes (4%; *p*-value <1.5e-08, Chi-squared test), we decided to concentrate our analysis on the functional relationship between PABPN1 and the upregulated lncRNAs. We first independently validated the results obtained by RNA-seq. Northern blot and quantitative RT-PCR analyses were used to confirm the accumulation of transcripts that belong to different classes of lncRNAs, including snoRNA- and scaRNA-host transcripts (Figure 3A–3B), previously described lncRNAs (Figure 3C–3D, NEAT1 and TUG1, respectively), as well as putative lncRNAs (Figure 3E). In the case of noncoding snoRNA-host transcripts, we also confirmed that the levels of mature snoRNA (Figure 3A, SNORD60) and of the unspliced SHG60 transcript (data not shown) were unchanged in PABPN1–depleted cells, consistent with the RNA-seq data (Figure 2C).

We next examined the genomic environment of genes encoding PABPN1–senstitive lncRNAs. Interestingly, we found that lncRNA genes upregulated in PABPN1–depleted conditions are concentrated near transcription start sites (TSS) of neighboring protein-coding genes (Figure S4). Significantly, of the 60 lncRNAs upregulated in cells deficient for PABPN1, 24 are within 5-Kb upstream of a TSS, whereas only one lncRNA is found within 5-Kb downstream of a TSS (Table S4; *p*-value <0.001, Fisher's exact test). Based on these findings, we used strand information obtained from the gene annotations to examine whether lncRNAs upregulated in PABPN1–depleted cells were sense or antisense relative to the mRNA produced from the downstream-positioned genes. We found that the lncRNAs negatively controlled by PABPN1 are preferentially antisense relative to their downstream protein-coding genes: of the 24 lncRNAs located ≤5.0-Kb upstream of a transcription start site, 20 are in the antisense orientation. In addition, the fold changes in lncRNA expression induced by the loss of PABPN1 and the fold changes in the expression of their neighboring protein-coding gene are not correlated (Table S4), suggesting that the effect of PABPN1 on the control of lncRNA expression is specific. Our analysis also revealed that lncRNA regulation by PABPN1 was independent of transcript length and expression level (Figure S5).

Altogether, our transcriptome analysis indicates that a deficiency in PABPN1 does not globally affect mRNA expression, but revealed an important role for PABPN1 in the negative control of lncRNAs.

#### PABPN1 promotes the degradation of long noncoding RNAs

**Figure 4:**
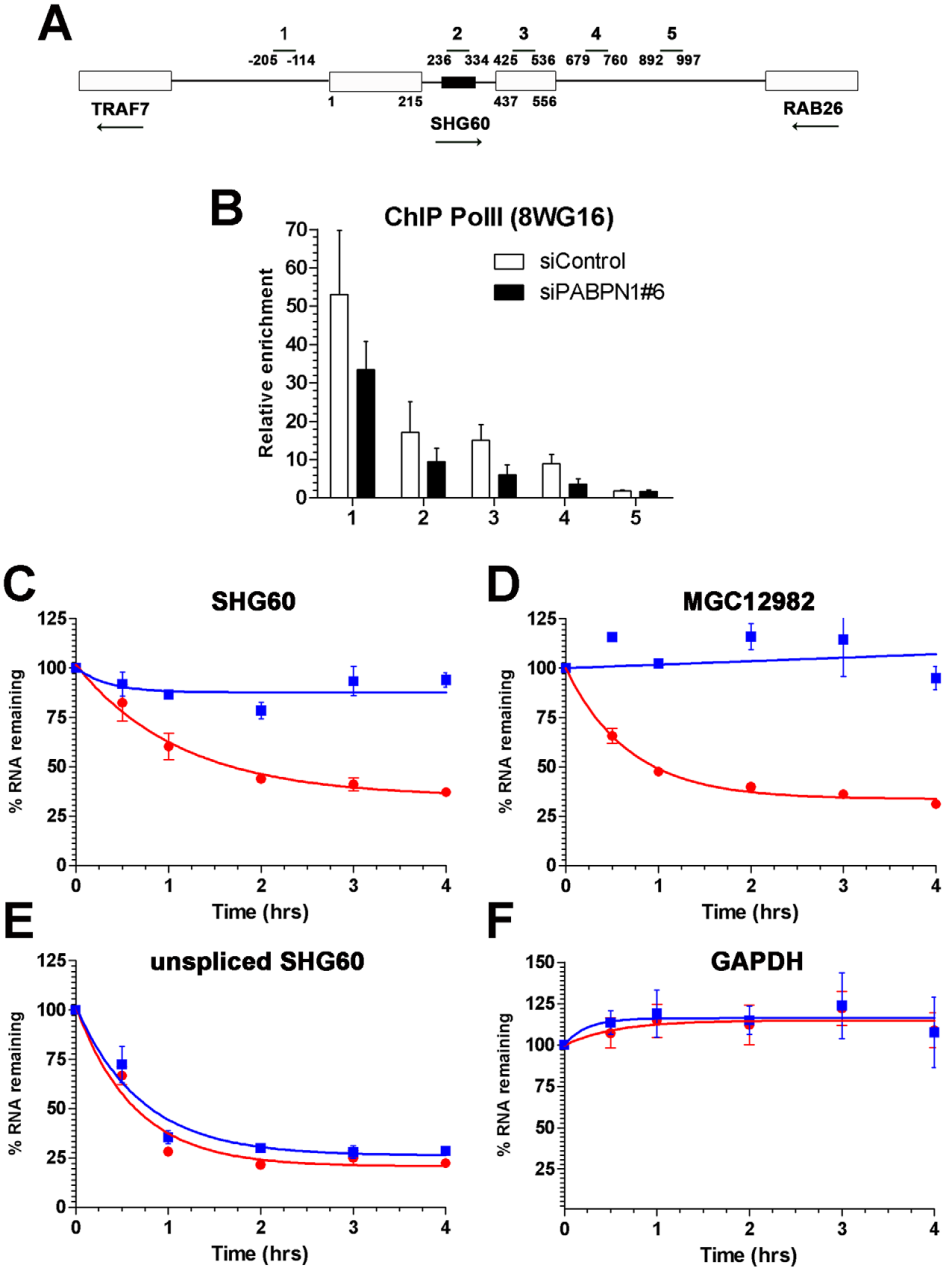
PABPN1 promotes lncRNA turnover. (A) Schematic diagram of the *SHG60* gene. White boxes represent exons and the small black box corresponds to the SNORD60 snoRNA located in the *SHG60* intron. The *TRAF7* and *RAB26* protein-coding genes located upstream and downstream, respectively, of SHG60 are also shown. Nucleotides numbers are relative to the first nucleotide from exon 1 of *SHG60*. Bars above the gene show the position of PCR products used for analyses in ChIP assays and for identification in panel B. (B) ChIP assays were performed on extracts prepared from cells treated with control (white bars) and PABPN1–specific (black bars) siRNAs using a monoclonal antibody (8WG16) specific to RNA Pol II. The coprecipitating DNA was quantified by real-time PCR using gene-specific primer pairs located along the *SHG60* gene (see panel A). ChIP data are presented as the fold enrichment of RNA Pol II relative to control purifications performed without antibody. Values represent the means of at least three independent experiments and bars correspond to standard deviations. (C–F) HeLa cells previously transfected with control (red circles) and PABPN1–specific (blue squares) siRNAs were treated with 5 µg/ml actinomycin D, and RNA was isolated at time zero and intervals thereafter indicated. RNA decay rates of the spliced SHG60 lncRNA (C), MGC12982 lncRNA (D), unspliced SHG60 transcripts (E), and GAPDH mRNA (F) were determined by quantitative RT-PCR analysis and normalized to the PABPC1 mRNA. The data and error bars represent the average and standard deviation from three independent experiments.

The observation that a PABPN1 deficiency caused the upregulation of spliced, but not of unspliced lncRNAs (Figure 2C) suggested a mechanism of posttranscriptional regulation. To confirm that the accumulation of lncRNAs in PABPN1–depleted cells was not the consequence of upregulated transcription, we examined the levels of RNA polymerase II (Pol II) by chromatin immunoprecipitation (ChIP) along the SHG60 gene (Figure 4A), which encodes the lncRNA showing the greatest upregulation in conditions of PABPN1 deficiency (Figure 2, Figure 3, Table S3). Pol II occupancy along the SHG60 gene was calculated using the input-normalized signal of targeted regions (Figure 4A) in Pol II precipitates relative to a control ChIP without antibody. SHG60 transcription was not upregulated in PABPN1–depleted cells (Figure 4B); in fact, Pol II levels were somewhat lower in conditions of PABPN1 deficiency relative to control cells. The decreasing trend in Pol II signal along SHG60 is consistent with ChIP data using the 8WG16 antibody [32], [33], which reacts primarily with the hypophosphorylated form of RNA Pol II that concentrates near gene promoters. A similar decreasing trend from 5′ to 3′ was observed using the 8WG16 antibody for a gene whose expression was not affected by PABPN1 knockdown (Figure S6A). We have also analyzed SHG60 transcription using a phospho-specific (serine 2) antibody to the large subunit of RNA Pol II and found similar levels of Pol II occupancy in PABPN1–depleted and controls cells (Figure S6B). Altogether, the results from these Pol II ChIP experiments suggest that the accumulation of SHG60 lncRNAs in PABPN1–depleted cells occurs primarily at the post-transcriptional level.

We therefore examined the effect of PABPN1 deficiency on lncRNA stability. Actinomycin D was used to inhibit transcription in PABPN1–depleted and controls cells, and RNA decay was followed over time using the GAPDH mRNA as a control. Depletion of PABPN1 clearly stabilized the spliced SHG60 lncRNA (Figure 4C, blue squares), consistent with its upregulation in PABPN1–depleted cells (Figure 2, Figure 3). A similar degree of stabilization was observed for the lncRNA expressed from the MGC12982 gene (Figure 4D), whose expression was substantially increased in PABPN1–depleted cells (Figure 3E; Table S3). In contrast, the stability of the unspliced SHG60 transcript (Figure 4E) and of the GAPDH mRNA (Figure 4F) was not affected in PABPN1 knockdown conditions. We thus conclude that the accumulation of lncRNAs in PABPN1–depleted cells is the consequence of RNA stabilization.

#### PABPN1–mediated control of lncRNA expression is polyadenylation-dependent

**Figure 5:**
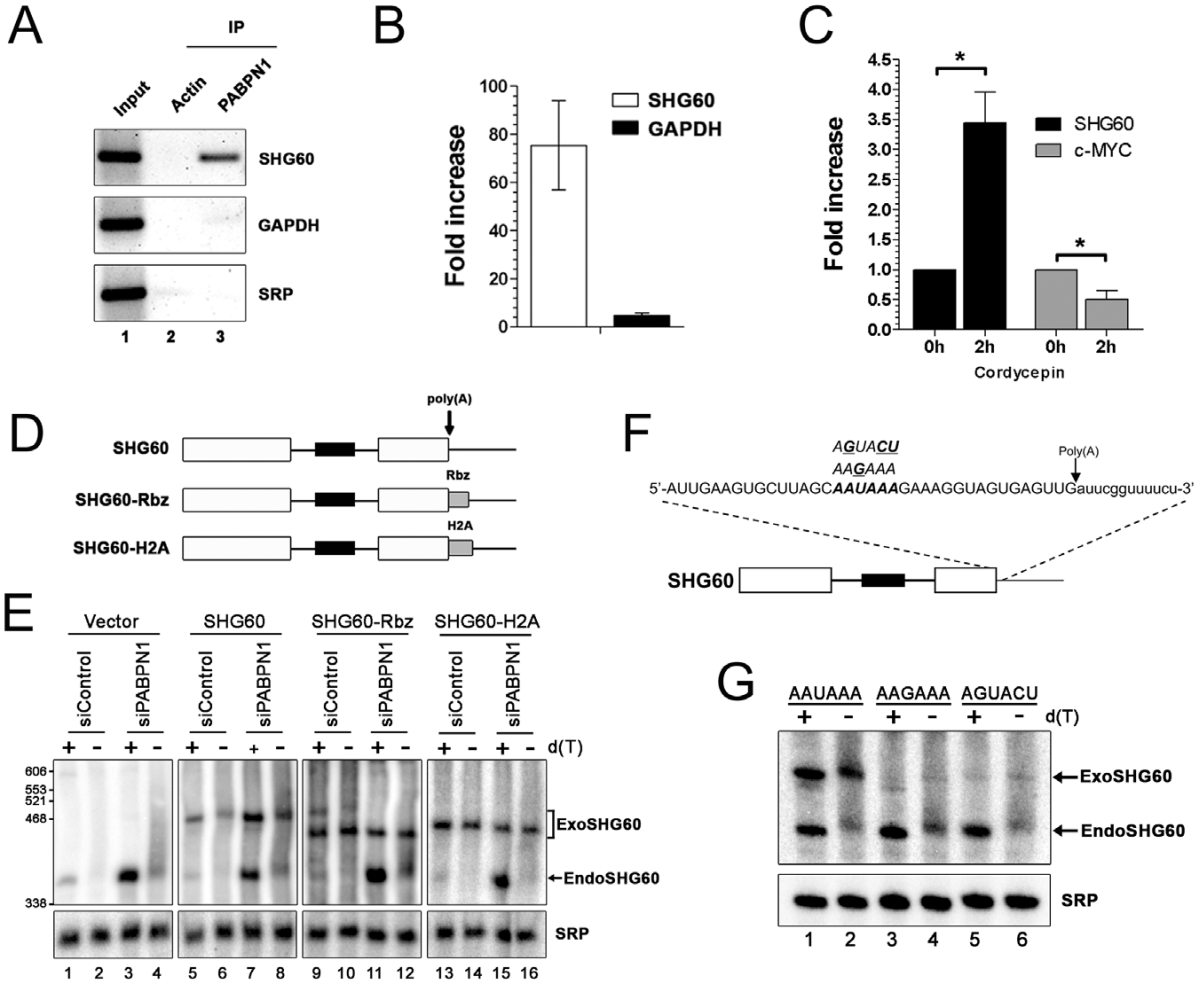
PABPN1–dependent RNA decay is polyadenylation-dependent. (A) Immunoprecipitation (IP) experiments showing that the SHG60 lncRNA is enriched in PABPN1 precipitates, but not in a control purification. The GAPDH mRNA and the SRP noncoding RNA were used as controls. (B) RNA enrichments (IP∶input ratio) in PABPN1 and control purifications were determined by real-time RT-PCR for the SHG60 RNA, the GAPDH mRNA, and the SRP RNA. Fold changes are relative to the control actin IP and normalized to the SRP RNA. The data and error bars represent the average and standard deviation from four independent experiments. (C) Quantitative RT-PCR analysis of RNA prepared from HeLa cells that were previously treated or not treated with cordycepin for 2 h. Fold increases are relative to untreated cells and normalized to the nonpolyadenylated SRP RNA. The data and error bars represent the average and standard deviation from three independent experiments. * *p*<0.05; Student's t-test. (D) Schematic diagram of the SHG60 constructs. White rectangles represent the noncoding exons, whereas the black box corresponds to the intronic SNORD60. The arrow indicates the position of the polyadenylation site as determined by 3′ RACE. The RBZ and H2A grey boxes correspond to the ribozyme and H2A terminator sequences, respectively. (E) HeLa cells treated with PABPN1–specific and control siRNAs were transfected with the control vector (lanes 1–4) or DNA constructs that express normal (lanes 5–8), ribozyme-processed (lanes 9–12), and H2A-processed (lanes 13–16) SHG60 lncRNA. Total RNA was treated with RNase H in the presence (+) or absence (−) of oligo(dT) before northern analysis using a SHG60-specific probe. The position of exogenous and endogenous SHG60 lncRNA is indicated on the right. The SRP RNA was used as a loading control. (F) The AAUAAA hexamer sequence of the SHG60 poly(A) signal is shown in bold with the two different hexamer variants used shown above the AAUAAA hexamer. The arrow shows the position of the SHG60 polyadenylation site, as determined by 3′ RACE. (G) HeLa cells were transfected with DNA constructs that express AAUAAA (lanes 1–2), AAGAAA (lanes 3–4), and AGUACU (lanes 5–6) SHG60 lncRNA. Endogenous and exogenous SHG60 lncRNA were detected in PABPN1–expressing conditions as shown in E (lanes 5–6).

To address whether PABPN1 directly regulates the expression of lncRNAs, RNA co-immunoprecipitation (IP) experiments were used to examine whether PABPN1 can bind to lncRNAs. As can be seen in Figure 5A, the SHG60 lncRNA was clearly enriched in a PABPN1 precipitate as compared to a control purification (upper panel, lanes 2–3). In contrast, no enrichments were detected for the abundant GAPDH mRNA and the non-polyadenylated SRP RNA using similar RT-PCR conditions (Figure 5A, middle and bottom panels, respectively). No signal was detected in the absence of reverse transcription, indicating that the observed amplification was not due to the presence of residual DNA in the immunoprecipitate (data not shown). A quantitative analysis of the RNA-IP experiments using real-time RT-PCR indicated that the GAPDH mRNA was enriched by 5–8–fold in PABPN1 precipitates relative to a control nonpolyadenylated RNA (Figure 5B). Notably, we found that the enrichment level of the SHG60 lncRNA in the PABPN1 purification was about 20 times greater than for the GAPDH mRNA (Figure 5B), which is consistent with our qualitative RT-PCR analysis (Figure 5A). These results indicate that a significantly greater (*p*-value <0.02, Student's *t*-test) proportion of SHG60 lncRNA is bound by PABPN1 relative to the GAPDH mRNA population.

Gene regulation by a nuclear poly(A)-binding protein at the level of lncRNA decay predicts a polyadenylation-dependent mechanism. To test this prediction, we first analyzed the effect of cordycepin, which causes premature termination of poly(A) tail synthesis by inhibiting the poly(A) polymerase [34], on the expression of the SHG60 lncRNA. Notably, the SHG60 lncRNA was significantly stabilized in cordycepin-treated cells (Figure 5C), consistent with a polyadenylation-dependent mechanism of RNA decay. As a control, inhibition of polyadenylation by cordycepin resulted in decreased levels of the unstable c-MYC mRNA (Figure 5C), consistent with previous results [35]. To get further evidence about the direct role of PABPN1 in polyadenylation-dependent lncRNA turnover, we next addressed whether a 3′ poly(A) tail was required for PABPN1–dependent control of SHG60 lncRNA expression. A construct was made in which a variant of the hepatitis Delta ribozyme was inserted upstream of the SHG60 polyadenylation signal (Figure 5D). The hepatitis Delta ribozyme was chosen as it was previously shown to promote the synthesis of non-polyadenylated transcripts in human cells [36]. Normal and ribozyme-containing SHG60 constructs were transiently transfected in HeLa cells that were previously treated with control and PABPN1–specific siRNAs. Consistent with our previous results, the endogenous SHG60 lncRNA was upregulated in PABPN1–depleted cells (Figure 5E, lanes 1–4). To assess the polyadenylation status of the SHG60 lncRNA, a fraction of total RNA was treated with RNase H in the presence of oligo d(T), which causes the heterogeneous polyadenylated products (lanes 2 and 4) to collapse into the expected 355-nt-long spliced lncRNA (lanes 1 and 3). On the basis of these RNase H assays, we estimated that the median poly(A) tail of the spliced SHG60 lncRNA accumulating in PABPN1–depleted cells was ∼150-nt (lanes 3 and 4). Because the DNA constructs express the SHG60 lncRNA with additional plasmid sequences at the 5′ end, we were able to distinguish between the plasmid-expressed and endogenous SHG60 lncRNA using polyacrylamide gels (Figure 5E, lanes 5–8). As for endogenous SHG60, loss of PABPN1 resulted in the accumulation of polyadenylated exogenous SHG60 lncRNA expressed from the plasmid construct (compare lane 7 to 5). We confirmed that the SHG60 lncRNA expressed from the ribozyme construct was not polyadenylated, as a single product was detected with or without the addition of oligo d(T) (Figure 5E, compare lanes 9–10 and 11–12). Notably, the non-polyadenylated SHG60 transcript expressed from the ribozyme construct was largely insensitive to PABPN1–dependent degradation (Figure 5E, lanes 9–12). Similar results were obtained when we fused the SHG60 lncRNA to terminator sequences from the *H2A* histone gene, which encodes a non-polyadenylated mRNA (Figure 5D and Figure 5E, lanes 13–16). These results strongly support a direct role for PABPN1 in the turnover of the SHG60 lncRNA via a polyadenylation-dependent mechanism.

We also examined whether 3′ end processing of the SHG60 lncRNA involves *cis* elements normally required during mRNA 3′ end processing. Accordingly, we tested whether the AAUAAA hexamer found 16-nt upstream of the SHG60 lncRNA poly(A) site was required for SHG60 expression by transfecting HeLa cells with DNA constructs in which the AAUAAA hexamer was mutated to AAGAAA and AGUACU (Figure 5F). As was seen in Figure 5E, northern blot analysis detected the plasmid-expressed wild-type SHG60 lncRNA, which can be distinguished from the faster-migrating endogenous SHG60 lncRNA (Figure 5G, lanes 1–2). Notably, in cells transfected with the SHG60-*AAGAAA* and SHG60-*AGUACU* DNA constructs, the steady state level of exogenous SHG60 lncRNA was substantially reduced relative to wild-type SHG60 (Figure 5G, compare lanes 3–6 to lanes 1–2), consistent with defective 3′ end processing for both SHG60-*AAGAAA* and SHG60-*AGUACU* variants. These results indicate that the conserved AAUAAA sequence of the SHG60 poly(A) signal is required for proper 3′ end processing and suggest that PABPN1–induced lncRNA destabilization is coupled to poly(A) signal-mediated 3′ end processing.

#### The RNA exosome controls the expression of PABPN1–sensitive lncRNAs

**Figure 6:**
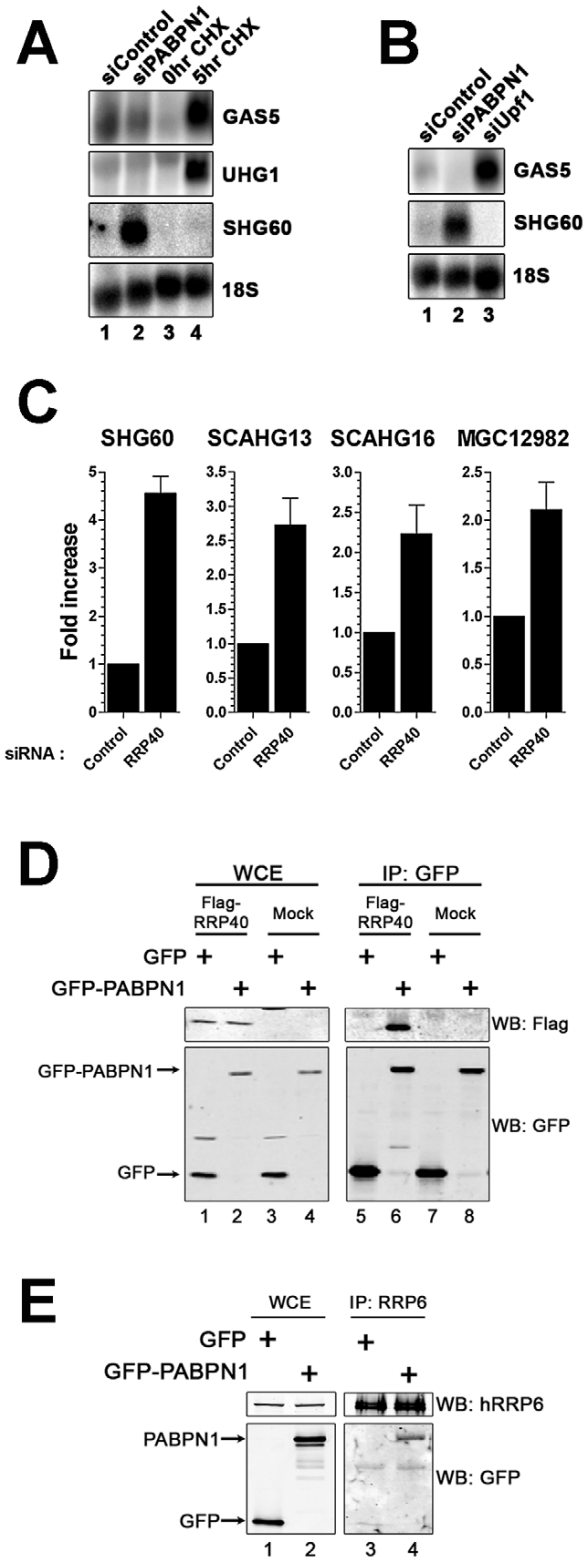
The RNA exosome, but not nonsense-mediated decay, contributes to PABPN1–dependent lncRNA decay. (A) Northern blot analysis of RNA prepared from untreated (lane 3) and cycloheximide-treated (lane 4; 20 µg/ml) HEK293T cells, as well as from cells treated with PABPN1–specific (lane 2) and control (lane 1) siRNAs. The blot was probed for the GAS5, UHG1, and SHG60 noncoding transcripts. The 18S rRNA was used as a loading control. (B) Northern analysis of RNA prepared from cells treated with control (lane 1), PABPN1–specific (lane 2), and UPF1-specific (lane 3) siRNAs. The blot was probed for the GAS5, SHG60, and 18S RNAs. (C) Quantitative RT-PCR analysis of RNA prepared from HeLa cells treated with control and hRRP40-specific siRNAs using sequence-specific primers to the indicated genes. Fold increases are relative to control siRNA and normalized to GAPDH mRNA. The data and error bars represent the average and standard deviation from at least three independent experiments. (D) Western analysis of whole cell extract (WCE; lanes 1–4) and GFP immunoprecipiates (IP; lanes 5–8) prepared from HEK293T cells that stably express GFP (lanes 1, 3, 5, and 7) or GFP-PABPN1 (lanes 2, 4, 6, and 8) and that were previously transfected with the empty vector (lanes 3–4 and 7–8) or a construct that expressed a Flag-tagged version of the hRRP40 (lanes 1–2 and 5–6). Western analysis was performed using antibodies specific to Flag (upper panel) and GFP (bottom panel). (E) Western analysis of whole cell extract (WCE; lanes 1–2) and hRRP6 immunoprecipiates (IP; lanes 3–4) prepared from HEK293T cells that stably express GFP (lanes 1 and 3) and GFP-PABPN1 (lanes 2 and 4). Western analysis was performed using antibodies specific to endogenous hRRP6 (upper panel) and GFP (bottom panel).

The observation that the accumulation of lncRNAs in PABPN1–depleted cells was associated with RNA stabilization (Figure 4) suggested that PABPN1 might promote an RNA decay pathway. Similar to SHG60, the GAS5 and UHG1 snoRNA-host genes produce spliced and polyadenylated lncRNAs that are almost undetectable in normal growing cells [37]. Because translation inhibition results in the accumulation of GAS5 and UHG1 lncRNAs [38], we examined whether translation inhibition using cycloheximide causes the accumulation of the SHG60 lncRNA. Whereas the GAS5 and UHG1 transcripts clearly accumulated after translation inhibition (Figure 6A, lanes 3–4), the expression level of the SHG60 lncRNA was not increased (Figure 6A, lanes 3–4). In addition, the SHG60 lncRNA did not accumulate after the depletion of a key component of nonsense-mediated RNA decay (NMD), UPF1 (Figure 6B, lane 3), consistent with a translation-independent mechanism of RNA decay. In contrast, the GAS5 lncRNA was sensitive to NMD inactivation (Figure 6B, lane 3). These results indicate that PABPN1 promotes the degradation of the SHG60 lncRNA by a mechanism independent of NMD.

In fission yeast, the PABPN1 homolog physically associates with the exosome complex of 3′→5′ exonucleases to promote RNA degradation [8], [18], [19], [20]. We therefore examined the effect of depleting a component of the RNA exosome on the expression level of the SHG60 lncRNA. We chose to deplete hRRP40, a core component of the exosome, as the individual depletion of hRRP40 was previously shown to stabilize other noncoding RNAs [39]. siRNAs specific to the *RRP40* mRNA depleted ∼80% of the total hRRP40 protein (Figure S7) and stabilized the SHG60 lncRNA as well as different PABPN1–sensitive lncRNAs (Figure 6C), suggesting that the RNA exosome is involved in the turnover of PABPN1–regulated lncRNAs. In contrast, depletion of XRN2 and PARN, other factors involved in nuclear RNA turnover, had no effect on SHG60 lncRNA levels (Figure S8). The functional role of hRRP40 in the regulation of these lncRNAs prompted us to examine whether PABPN1 associates with the human exosome complex. To test this possibility, we expressed a Flag-tagged version of hRRP40 in HEK293T cells that stably express GFP or GFP-PABPN1. Notably, we observed that immunoprecipitation of PABPN1 also pulled down hRRP40 (Figure 6D, lane 6); in contrast, precipitates from extracts of control cells did not (lane 5). We also used an affinity-purified antibody specific to hRRP6, a component of the nuclear exosome [40], to immunoprecipitate endogenous hRRP6 from cell extracts. As can be seen in Figure 6E, PABPN1 was specifically copurified with endogenous hRRP6 (lane 4), whereas the GFP control was not (lane 3). These results support the involvement of the RNA exosome in PABPN1–dependent lncRNA turnover.

**Figure 7:**
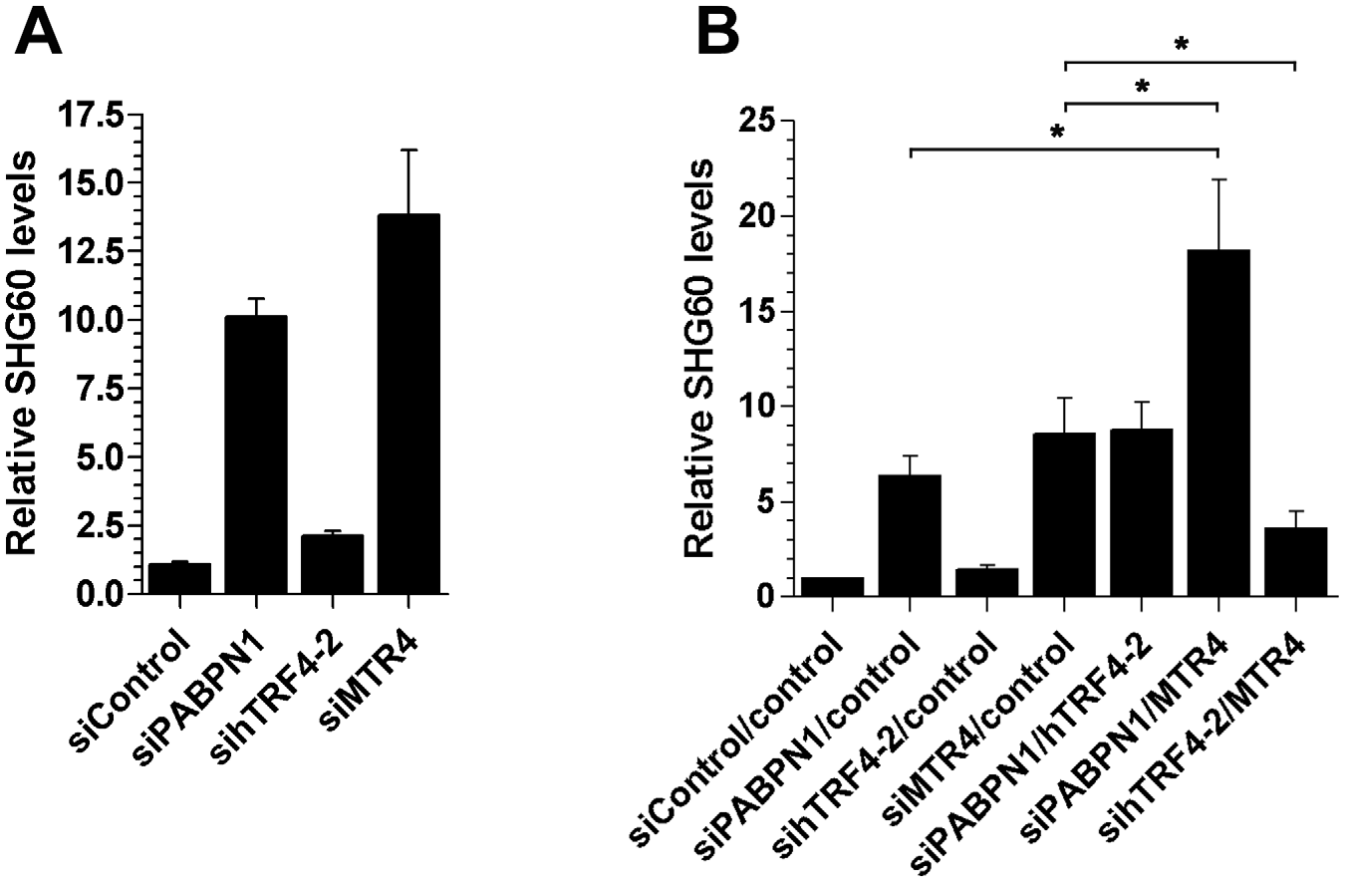
Efficient turnover of a PABPN1–sensitive lncRNA does not require hTRF4-dependent polyadenylation. (A) Total RNA from HeLa cells treated with the indicated siRNAs was analyzed by quantitative RT-PCR using primer pairs specific to SHG60. SHG60 lncRNA expression is relative to control siRNA and normalized to the GAPDH mRNA. The data and error bars represent the average and standard deviation from at least three independent experiments. (B) Total RNA from HeLa cells treated with the indicated combinations of siRNAs was analyzed by quantitative RT-PCR using primer pairs specific to SHG60. SHG60 expression is relative to cells treated with the combination of non-targeting siRNAs (control/control) and normalized to the GAPDH mRNA. The data and error bars represent the average and standard deviation from at least three independent experiments. (* *p*<0.05; Student's t-test).

In yeast, a specialized RNA polyadenylation complex, called TRAMP, can promote the degradation activity of the nuclear exosome for a variety of target RNAs [41]. Although the existence of a similar TRAMP-like complex in human cells remains elusive, a recent study reported physical interactions between the human exosome and putative TRAMP subunits [42]. We therefore used siRNA-mediated gene silencing to examine whether putative human TRAMP components were required for the negative regulation of the SHG60 lncRNA. We choose to target a catalytic subunit of the TRAMP complex, hTRF4-2, which is involved in the adenylation of RNA degradation products [42], [43] as well as the RNA helicase hMTR4 (SKIV2L2), which promotes exosome-mediated decay of specific noncoding RNAs [42]. We found only modest SHG60 lncRNA accumulation in conditions of hTRF4-2 deficiency (Figure 7A). In contrast, depletion of hMTR4 resulted in levels of SHG60 lncRNA accumulation similar to that of PABPN1 deficiency (Figure 7A), suggesting that hMTR4 contributes to the efficient turnover of the SHG60 lncRNA. To further characterize the relationship between PABPN1 and these human TRAMP subunits, we examined the effect of co-depleting PABPN1 with either hTRF4-2 or hMTR4. Whereas co-depletion of PABPN1 and hTRF4-2 did not significantly affect the levels of SHG60 lncRNA relative to the single PABPN1 depletion (Figure 7B), co-depletion of PABPN1 and hMTR4 resulted in a significant increase in SHG60 lncRNA levels relative to either single depletion (Figure 7B). Interestingly, we also noticed that co-depletion of MTR4 and hTRF4-2 suppressed the accumulation of SHG60 lncRNA detected in the single MTR4 depletion (Figure 7B). The reduced SHG60 signal in conditions of MTR4/TRF4-2 co-depletion relative to the single MTR4 depletion cannot be simply explained by the expression of hypoadenylated lncRNAs, as RT-PCR analyses were primed using random hexamers. Similar results were observed between hTRF4-2 and hMTR4 as well as between hTRF4-2 and RRP40 when a different class of substrate RNA, promoter upstream transcripts (PROMPTs), were analyzed (Figure S9; [42]). Altogether, our data suggest that PABPN1 and hMTR4 both contribute to exosome-mediated degradation of the SHG60 lncRNA.

## Discussion

The poly(A)-binding protein nuclear 1 (PABPN1) is required for efficient and processive poly(A) tail synthesis *in vitro*. Consequently, PABPN1 has generally been assumed to be required for mRNA synthesis, as RNA polyadenylation is a fundamental step of gene expression. Here we analyzed the transcriptome of PABPN1–deficient cells to address the functional role of PABPN1 in human gene expression. In contrast to the expected role of PABPN1 in general mRNA metabolism, we found that the expression level of most protein-coding genes was not affected in cells deficient for PABPN1. Surprisingly, the loss of PABPN1 resulted in the accumulation of several long noncoding RNAs (lncRNAs), as a consequence of their stabilization. Our findings thus elucidate a novel function for PABPN1 in the post-transcriptional control of noncoding RNAs.

### PABPN1 deficiency does not globally affect human gene expression

The biochemical characterization of mammalian PABPN1 has led to a model in which PABPN1 is critical for mRNA polyadenylation, and therefore gene expression [1], [3], [44]. The data presented in this study, however, are not consistent with a general role for PABPN1 in mRNA maturation. First, knockdown of PABPN1 using independent siRNAs did not inhibit the expression of a handful of protein-coding genes, as one might expect due to the lack of mRNA polyadenylation. Second, 3′ end analysis of specific mRNAs did not reveal a substantial reduction of polyadenylation activity in PABPN1–depleted cells. Third, and more importantly, we could deplete >90% of human PABPN1 without affecting the abundance of most mRNAs, as revealed by our RNA sequencing analysis. Although our data suggesting that PABPN1 is not required for the bulk of mRNA expression echoes findings in fission yeast [8] and *C. elegans* [9], this conclusion is not entirely supported by other studies. Accordingly, depletion of PABPN1 in mouse primary myoblasts results in a moderate, but significant poly(A) tail shortening using total RNA or individual mRNAs [6]. Poly(A) tail shortening has also been noted for specific *Drosophila pabp2* mutants [7]. Yet, the global impact of such poly(A) tail shortening on gene expression was not clearly investigated in these studies [6], [7]. Therefore, although there may be specific cell types that are highly dependent on PABPN1 for mRNA polyadenylation, we found that the loss of PABPN1 in independent human cellular systems did not result in a general inhibition of gene expression. Our findings argue that the activities attributed to PABPN1 on the basis of its biochemical characterization *in vitro*, namely stimulation of poly(A) polymerase processivity and poly(A) tail length control, may not necessarily apply to all genes and all cell types, suggesting that the mechanism underlying nuclear poly(A) tail synthesis is likely to be more complex than previously anticipated.

Our transcriptome analysis revealed that only ∼4% of the expressed protein-coding genes were misregulated in PABPN1–depleted cells. While the mechanism by which PABPN1 controls – directly or indirectly – the expression of specific protein-coding genes remains to be determined, such mRNA-specific regulation is apparently not associated to the recently described role of PABPN1 as a regulator of polyadenylation site decision [16], [45]. Indeed, we found no significant overlap between the set of genes controlled by PABPN1 at the level of 3′ end processing [16], [45] and the genes identified in our study that showed increased and decreased mRNA levels in PABPN1–depleted cells. This absence of overlap is consistent with the view that PABPN1–dependent control of polyadenylation site decision primarily affects microRNA-mediated translational regulation of targeted genes, rather than mRNA turnover [16]. Accordingly, Jenal et al. [16] found no change in the stability (i.e. expression) of several mRNAs (short and long isoforms) that showed a strong shift in polyadenylation site usage in conditions of PABPN1 deficiency. Consistent with these results, we also observed a tendency for 3′ UTR shortening for a subset of genes that have been shown to present a switch in poly(A) site usage upon depletion of PABPN1 [16], and found no significant changes in mRNA levels as measured by RNA-seq and by independent qRT-PCR assays (Figure S10). Thus, although alternative polyadenylation site usage can affect mRNA turnover, this does not appear to be necessarily the case for genes targeted by PABPN1. It should also be noted that previous works [16], [45] used sequencing technologies specifically developed to map the 3′ end of transcripts, which are not well suited to measure overall mRNA expression [46]. Our approach, on the other hand, is devised to accurately measure changes in gene expression [46]. Therefore, the two approaches should be regarded as complementary, since the aspects of gene expression and mRNA 3′ end processing studied are different.

### A function for PABPN1 in the control of lncRNA expression

A surprising finding from the analysis of our RNA sequencing results was the impact of PABPN1 deficiency on the human noncoding transcriptome. Accordingly, our analysis revealed that a significant proportion (∼16%) of the expressed long noncoding (lnc) RNA genes were misregulated in PABPN1–deficient cells. Notably, the majority (78%) of lncRNAs misregulated in PABPN1–deficient cells showed increased expression, a result that supports a more prominent role for PABPN1 in the negative control of lncRNA expression. The past few years have seen increasing interest in the identification and characterization of mammalian lncRNAs [25], [47]. lncRNAs have been shown to play key roles in different biological processes, acting via interactions with chromatin-modifying complexes [48], [49], [50], competing with mRNAs for microRNA binding sites [51], [52], [53], [54], [55], facilitating the recruitment of RNA degradation pathways [56], and acting as decoy for specific transcription [57] and splicing factors [58]. Yet, despite substantial progress into the functional characterization of lncRNAs, little is known about mechanisms that control their expression. Data from transcriptome profiles indicate that human lncRNAs are expressed in a highly tissue-specific manner [26], [59]. This observation suggests that mechanisms controlling lncRNA expression will significantly contribute to their functional diversity, allowing select lncRNAs to function in specialized tissues and cell types. Posttranscriptional gene regulation will likely be important to control the expression of mammalian lncRNAs, as demonstrated by the extensive range of decay kinetics recently determined for a large number of mouse lncRNAs [60]. Our study thus extends these recent reports by revealing a new pathway that controls human lncRNA turnover. Given the challenge of annotating lncRNA genes coupled to their tissue-specific nature, we speculate that the number of PABPN1–regulated lncRNAs identified in this study represents an underestimate.

Our data support the involvement of the nuclear exosome as a degradation machinery that catalyzes the turnover of PABPN1–sensitive lncRNAs (Figure 6). It is now established that poly(A) tail addition not only provides stability to eukaryotic RNAs, but also contributes to RNA turnover in the nucleus. This polyadenylation-dependent mechanism of nuclear RNA decay is mainly orchestrated by the exosome complex of 3′→5′ exonucleases. Although the poly(A) polymerase activity of the TRAMP complex is believed to mark the bulk of RNAs targeted for exosome-mediated decay [21], [61], our results suggest that the function of PABPN1 in promoting lncRNA decay occurs independently of TRAMP-mediated polyadenylation. First, although the biochemical properties of a human “TRAMP-like” complex [42] await further characterization, the ∼150-nt-long poly(A) tails detected on the SHG60 lncRNA that accumulated in PABPN1–depleted cells (Figure 5) strikingly contrasts to the 3–5 adenosines added by the yeast TRAMP complex [62], [63], [64]. Second, PABPN1–dependent control of lncRNA expression appears redundant to MTR4/SKIVL2, a protein associated to a putative human TRAMP complex [42], as co-depletion of PABPN1 and MTR4 resulted in additive SHG60 lncRNA accumulation (Figure 7). Moreover, because the poly(A) polymerase activity of hTRF4-2 was not required for SHG60 lncRNA decay (Figure 7), MTR4 apparently promotes RNA degradation in the absence of other TRAMP subunits, a situation previously observed in yeast [65], [66], [67] and humans [42]. We thus favor a model in which PABPN1 promotes exosome-mediated decay of lncRNAs via the polyadenylation activity of the canonical cleavage/polyadenylation machinery, a complex that contains PABPN1 [68]. Such a model for PABPN1–dependent RNA decay is remarkably similar to how its fission yeast homolog, Pab2, activates the degradation of specific transcripts in *S. pombe*. Accordingly, Pab2 promotes TRAMP-independent, exosome-mediated decay of select transcripts via the polyadenylation activity of the canonical poly(A) polymerase [17], [18], [19], [20], suggesting that this polyadenylation-dependent RNA decay pathway has been conserved.

Although the exact features that distinguish PABPN1–sensitive lncRNAs from other polyadenylated noncoding transcripts await further investigation, we found that lncRNAs stabilized in PABPN1–depleted cells were frequently located near and upstream of transcription start sites of neighboring coding genes. The existence of noncoding transcripts produced in the vicinity of transcription start sites of protein-coding genes has previously been observed in metazoans [39], [69], [70]. A specific class of such noncoding RNAs, promoter upstream transcripts (PROMPTs), has in fact been shown to be targeted by the human exosome [39], [71]. In contrast to PROMPTs, however, several PABPN1–sensitive lncRNAs correspond to spliced transcripts. Furthermore, whereas PROMPTs have been proposed to be associated to the promoter of most actively transcribed human genes [39], we did not find such a general trend for PABPN1–sensitive lncRNAs. In fact, depletion of PABPN1 did not significantly perturb the expression pattern of several previously described PROMPTs (Figure S11). Thus, despite a potential link with regard to how transcription of PROMPTs and PABPN1–sensitive lncRNAs is initiated, our data support the existence of distinct pathways that can prepare specific RNA substrates for the human exosome, a conclusion consistent with the discovery of multiple exosome cofactors in yeast [41], [72].

### lncRNA and OPMD

Expanding numbers of human disease-associated lncRNAs have been reported in the past few years [73]. Although many PABPN1–sensitive lncRNAs, such as the spliced lncRNA expressed from the SHG60 gene, are normally short-lived due to PABPN1–dependent decay, it is likely that these noncoding RNAs will have functional roles under specific growth conditions. This is apparently the case for the GAS5 snoRNA host gene, which also produces a lncRNA that is unstable in cycling human cells [38]. In growth-arrested cells, however, the spliced GAS5 lncRNA accumulates and promotes apoptosis by inhibiting the transcriptional activity of the glucocorticoid receptor [57]. Similarly, the spliced form of another noncoding snoRNA-host transcript was recently shown to be important for cell differentiation in the mammary gland [74]. As PABPN1 expression levels vary considerably across human tissues, appearing exceptionally weak in skeletal and cardiac muscle cells [75], it will therefore be interesting to determine whether misregulation of PABPN1–sensitive lncRNAs contribute to the development of oculopharyngeal muscular dystrophy, which is caused by mutation in the *PABPN1* gene.

In sum, we have performed the first transcriptome-wide analysis of PABPN1–dependent gene expression in human cells. Our results are not consistent with a general role for PABPN1 in mRNA maturation, as the majority of protein-coding genes expressed normal mRNA levels in cells deficient for PABPN1. Importantly, our findings unveiled a new function for PABPN1 in the control of lncRNA expression and provide evidence for the evolutionarily conserved role of PABPN1 in polyadenylation-dependent RNA decay.

## Methods

### Cell culture

HEK293T and HeLa cell lines were grown in DMEM supplemented with 10% FBS. siRNAs were transfected with lipofectamine 2000 at a final concentration of 25 nM for 72 hrs. Cordycepin 5′-triphosphate (Sigma Aldrich C9137) was dissolved in water and added to the cell culture medium at a concentration of 20 µM.

### RNAi–mediated knockdowns

siRNAs are from Dharmacon/Thermo Scientific. Control siRNAs (ON-TARGETplus Non-targeting siRNA #1 and #4), siPABPN1#5 (GGAACGGCCUGGAGUCUGAUU), siPABPN1#6 (AGUCAACCGUGUUACCAUAUU), siUPF1 (GGAACCACCUGCUGAACUATT); sihRRP40 (CACGCACAGUACUAGGUCAdTdT), siMTR4 (C A A U U A A G G C U C U G A G U A A U U), sihTRF4-2 (G C G C U G A C G U C C A G A U A U U U U), siPARN (G G A A G A A G A A A G A C A G U U A U U), and siXRN2 (G A G U A C A G A U G A U C A U G U U U U).

### Expression constructs

pEGFP-PABPN1 was previously described [76] and was a generous gift from Dr Guy Rouleau (Université de Montréal). To express the SHG60 lncRNA on a plasmid, the *SGH60* gene (exon1-intron-exon2) plus 1-kb of downstream sequence was PCR-amplified using genomic DNA and cloned into pcDNA5.1. The *SGH60*-ribozyme and *SGH60*-H2A constructs were generated by inserting a variant of the hepatitis d ribozyme and terminator sequences from the histone *H2A* gene, respectively, 2-nt upstream of the *SGH60* polyadenylation site. The ribozyme and *H2A*-terminator sequences used were previously described [36], [77]. The SGH60-*AAGAAA* and SGH60-*AGTACU* poly(A) signal variants were generated by site-directed mutagenesis using the wild-type SHG60 construct. All of the constructs used in this study were verified by automated sequencing.

### Antibodies

The rabbit monoclonal antibodies specific to PABPN1 and Actin were obtained from Epitomics Inc.. A rabbit polyclonal anti-PABPN1 was also used in this study (a gift from Dr David Bear, University of New Mexico). The rabbit polyclonal antibody specific for hRRP6/EXOSC10 was from Abcam. The monoclonal antibody specific to RNA polymerase II (8WG16) was from Covance and the anti-RNA Pol II antibody specific for Serine-2 phosphorylated CTD repeats was from Abcam (Ab5095). The mouse monoclonal GFP and Flag antibodies were from Roche and Sigma, respectively.

### Library preparation for RNA–seq

Libraries were prepared from total human RNA and sequenced using the Illumina HiSeq technology. We obtained 196,998,210 and 192,880,594 100-bp paired-end reads for control and PABPN1–depleted cells, respectively (Table S5). Reads containing adapter sequences (>11 nt) were removed from the dataset, and low quality bases at read termini were trimmed using the fastx toolkit (http://hannonlab.cshl.edu/fastx_toolkit/index.html), discarding short reads (<32 nt) after trimming. Tophat [23] was used to perform a spliced alignment to the human reference genome build hg19. Integrative Genomics Viewer [78] was used for visualization.

### Expression analysis

A comprehensive set of noncoding RNA genes was compiled by combining annotations from different sources, which are specified in Table S6. When necessary, genomic coordinates were converted to hg19 coordinates using lift Genome at the UCSC genome browser [79]. An independent set of protein-coding genes was defined as all UCSC genes (Based on RefSeq, UniProt, GenBank, CCDS and Comparative Genomics) with associated protein ID (Uniprot, RefSeq protein or SwissProt), removing highly redundant genes (completely contained within another gene). A subset of protein-coding genes whose genomic coordinates do not overlap any of the previously annotated ncRNA genes was also compiled, thus excluding protein-coding genes that also harbor annotated ncRNA genes. To estimate gene expression levels, we used all exonic reads mapping within the maximal genomic locus containing each gene and its known isoforms, normalizing by the length of the mappable region and the total number of reads in the sample (RPKM, reads per kilobase of million reads). Fold changes were computed from normalized read counts for genes expressed above a minimum level (RPKM>1) in at least one of the samples. BEDTools [80] and custom scripts were used to perform these calculations. The expression levels obtained for all coding genes (including those overlapping ncRNA genes) can be found in Table S1, whereas the expression levels obtained for lncRNA genes are reported in Table S3.

Neighboring gene expression analysis: BEDTools and customs scripts were used to find, for each lncRNA gene, the closest transcription start site (TSS) of a protein-coding gene expressed above a minimum level (RPKM>1 in at least one of the samples). Strand information from current gene annotations was used to define transcripts as sense (the strand of the lncRNA equals the strand of the protein-coding gene) or antisense.

### Accession number

The Sequence Read Archive (SRA) number for the deep sequencing data reported in this paper is SRP015926.

### RNA analyses

Northern blot were hybridized using ^32^P-labeled gene-specific probes. Signals were detected and quantified using a Typhoon Trio instrument. Real-time quantitative RT-PCR analysis were as previously described [18]. Spliced versions of lncRNAs were analyzed using primers pairs in which one primer was complementary to an exon-exon junction.

### Chromatin immunoprecipitation assays (ChIP)

HeLa cells that were previously transfected with siRNAs against PABPN1/Control for 72 h, were washed and cross-linked for 10 min at 37°C by addition of formaldehyde at a final concentration of 1%. Cells were washed in PBS, resuspended in 100 µl of ChIP lysis buffer [1% sodium dodecyl sulfate (SDS), 10 mM EDTA, 50 mM Tris-HCl (pH 8.0), and protease inhibitors], and sonicated with a Branson Sonifier 450 at 20% amplitude with 10-sec pulses at 3 min cycle. The chromatin solution was diluted 10-fold in ChIP dilution buffer (0.01% SDS; 1.1% Triton X-100; 1.2 mM EDTA; 16.7 mM Tris, pH 8.1; 167 mM NaCl; and protease inhibitors). 5% of the chromatin extracts were used for purification of total DNA by ChIP. Each sample was precleared by incubating with 40 µl salmon sperm DNA/protein A-agarose 50% gel slurry for 2 hrs at 4°C. An aliquot of 4 µl of anti-RNA Polymerase II monoclonal Antibody (8WG16) was added and immunoprecipitated at 4°C overnight. The immunoprecipitates were collected using salmon sperm DNA/protein A-agarose and washed once with the following buffers in sequence: low-salt wash buffer (0.1% SDS; 1% Triton X-100; 2 mM EDTA; 20 mM Tri-HCl, pH 8.1; 150 mM NaCl); high-salt wash buffer (0.1% SDS; 1% Triton X-100; 2 mM EDTA; 20 mM Tris-HCl, pH 8.1; 500 mM NaCl); LiCl wash buffer (0.25 m LiCl; 1% Nonidet P-40; 1% sodium deoxycholate; 1 mM EDTA; 10 mM Tris-HCl, pH 8.1); and 3 times with TE (10 mM Tris-HCl, pH 8.0; 1 mM EDTA). DNA-protein cross-links were reversed by incubation at 65°C overnight followed by proteinase K treatment. DNA was recovered by purification with the Qiaquik PCR purification column (QIAGEN). Quantitative real-time PCR was performed using total DNA as control, and immunoprecipitated DNA. Fold enrichments of RNA Pol II are relative to control ChIPs performed without antibody, as we found identical results regardless of using IgG control (abcam ab46540) or no antibody.

### Immunoprecipitation experiments

Immunoprecipitation experiments were as previously described [81]. Cells were lyzed in buffer A (20 mM HEPES pH 7.9, 300 mM KCl, 10% glycerol, 0.5 mM DTT, 1 mM EDTA, 2 mM MgCl_2_, and 1% NP-40) for RNA coimmunoprecipitations and buffer B (50 mM Tris-HCl pH 7.5, 150 mM NaCl, 1% Triton-X-100, 10% glycerol, 2 mM MgCl_2_, 1 mM DTT) for protein coimmunoprecipitations. Total cell lysates were subjected to immunoprecipitation using specific antibodies and the antibody-coated beads were washed four times in lysis buffer before analyses by Western blot and RT-PCR. For the quantitative RT-PCR analysis of RNA IPs, fold changes were calculated relative to the control actin immunoprecipitation and normalized to the *SRP* RNA, as previously described [18].

## Supporting Information

Figure S1 lncRNAs are more affected than mRNAs by a deficiency in PABPN1. Histograms of log ratios/fold-change (FC) in expression levels (as determined using normalized RPKM counts of PABPN1–depleted conditions relative to control siRNA treatment) showing the distribution of (*A*) the 11,572 protein-coding genes and (*B*) the 469 lncRNA-coding genes detected RPKM >1 in at least one sample. The density distribution of these histograms was analyzed using Kernel density estimation statistics, yielding the smoothed distribution shown in Figure 2A.
(DOC)

Figure S2 The expression of lncRNA genes is more significantly affected by a deficiency in PABPN1 than protein-coding genes. Cumulative distribution of RPKM fold changes after depleting PABPN1. Plotted are distributions for the protein-coding (red) and lncRNA (blue) genes with RPKM >1. The number of genes in each category is indicated in parentheses. The two distributions are significantly different (Kolmogorov-Smirnov test p-value <2.2 e-16).
(DOC)

Figure S3 Genomic organization of the SNORD60 Host Gene (SHG60). The blue rectangular boxes represent the 2 exons of human SHG60; the green rectangle represents the SNORD60 snoRNA present in the SHG60 intron. Numbers above and below the gene are the lengths of exons and intron, respectively, as determined by 5′ and 3′ RACE experiments. Transcription of SHG60 produces a 555-nt-long unspliced precursor transcript. Splicing of the SHG60 precursor yields two products: (i) an excised intron that will be processed into the mature C/D box SNORD60 snoRNA, and (ii) a 335-nt-long polyadenylated RNA with limited coding potential (24-nt ORF shown in red).
(DOC)

Figure S4 lncRNA genes that are negatively regulated by PABPN1 are frequently located upstream and near the transcription start site of a neighboring protein-coding gene. Histogram showing the distribution of lncRNA genes up-regulated in PABPN1–depleted cells according to the distance from their nearest expressed protein-coding gene. Bin size: 10 Kb.
(DOC)

Figure S5 Negative regulation of lncRNA expression by PABPN1 is not correlated with RNA size and expression level. Scatter plots of RNA length (*A*, in nucleotides, *X*-axis) or normalized expression levels (*B*, number of reads, *X*-axis) versus the fold change in expression in PABPN1–depleted cells relative to control cells (*Y*-axis), for the 469 lncRNAs expressed >1 RPKM in at least one sample. Dots above the red horizontal line represent the 60 lncRNAs induced >2-fold in PABPN1–deficient cells.
(DOC)

Figure S6 PABPN1 depletion does not affect GAPDH and SHG60 transcription. ChIP assays were performed on extracts prepared from cells treated with control (white bars) and PABPN1–specific (black bars) siRNAs using (A) a monoclonal antibody (8WG16) specific to RNA Pol II and (B) an anti-RNA Pol II antibody specific for Serine-2 phosphorylated CTD repeats. The coprecipitating DNA was quantified by real-time PCR using gene-specific primer pairs located along the *GAPDH* gene (A) and *SHG60* gene (B). ChIP data are presented as percentage of input normalized to control purifications. Values represent the means of at least three independent experiments and bars correspond to standard deviations.
(DOC)

Figure S7 siRNA–mediated depletion of human RRP40. Western blot analysis of total extracts prepared from HeLa cells treated with RRP40-specific (lane 2) and control (lane 1) siRNAs for 72 hrs.
(DOC)

Figure S8 XRN2 and PARN are not required for the control of SHG60 lncRNA expression. Quantitative RT-PCR analysis of RNA prepared from HeLa cells treated with control siRNAs as well as with siRNAs specific to PABPN1, XRN2, and PARN. Fold increases in SHG60 lncRNA levels are relative to control siRNA and normalized to GAPDH mRNA. The data and error bars represent the average and standard deviation from at least three independent experiments.
(DOC)

Figure S9 Individual depletion of PABPN1 does not affect the expression of a promoter upstream transcript (PROMPT). Quantitative RT-PCR analysis of a representative PROMPT (40-33) region. HeLa cells were treated with the indicated combinations of siRNAs. Fold increases in 40-33 PROMPT levels are relative to the control/control siRNA mix and normalized to GAPDH mRNA. The data and error bars represent the average and standard deviation from at least three independent experiments.
(DOC)

Figure S10 A PABPN1–dependent switch in polyadenylation site usage does not necessarily induce a change in mRNA level. (A) Quantification of the fold changes in proximal/distal poly(A) site usage between PABPN1–depleted and control cells. Proximal/Distal ratios were calculated for the indicated genes using a previously described approach [84] in which 3′ UTRs are partitioned into two segments: a proximal 3′ UTR, delimited by sequences downstream of the STOP codon and upstream of the most proximal poly(A) site, and a distal 3′ UTR, delimited by sequences downstream of the proximal poly(A) site and upstream of the most distal poly(A) site. Coordinates for proximal and distal poly(A) sites were obtained from the poly(A) database [85]. The number of reads mapping to each of these segments was used to calculate RPKM values for proximal and distal 3′ UTRs for PABPN1–depleted and control samples. For eight of the nine tested genes that were previously shown to present a switch in poly(A) site usage upon depletion of PABPN1 [16], we observed increased usage of the proximal polyadenylation site in PABPN1–depleted conditions, in agreement with previous reports. Fold changes in gene expression as determined by RNA-seq are also indicated. (B) Examples of genes showing 3′ UTR shortening, indicated by the reduced level of read coverage downstream of the proximal poly(A) site relative to upstream the proximal poly(A) site in PABPN1–depleted cells compared to control cells. The red and blue arrows above the genes indicate their orientation. (C) Quantitative RT-PCR analysis of RNA prepared from HeLa cells that were treated with control and PABPN1–specific (#5 and #6) siRNAs using sequence-specific primers to the indicated genes. Fold increases are relative to control siRNA-treated cells and normalized to GAPDH mRNA. The SGH60 lncRNA was used as a positive control for PABPN1–dependent upregulation. The data and error bars represent the average and standard deviation from at least three independent experiments.
(DOC)

Figure S11 Loss of PABPN1 does not result in the accumulation of promoter upstream transcripts (PROMPTs). (A–D) Integrated Genome Viewer screenshots showing the transcriptional start sites of the C21orf63 (A), EXT1 (B), HMGN1 (C), and RBM39 (D) genes that were previously associated with PROMPT activity [39]. The coverage of the RNA-seq reads (grey) from cells treated with PABPN1–specific (si6; top) and control (NT4; bottom) siRNAs is shown along the RefSeq annotations (blue).
(DOC)

Table S1 Table showing all protein-coding genes with RPKM >1 in a least one sample. UCSC genes (Based on RefSeq, UniProt, GenBank, CCDS and Comparative Genomics), with a protein ID (Uniprot, RefSeq protein or SwissProt), after removal of redundant genes. Coding genes up-regulated and down-regulated >2-fold are shaded in green and orange, respectively.
(XLS)

Table S2 Table showing all protein-coding genes with RPKM >1 in a least one sample, not overlapping ncRNA genes, displaying more than 2 fold change in expression: UCSC genes (Based on RefSeq, UniProt, GenBank, CCDS and Comparative Genomics), with a protein ID (Uniprot, RefSeq protein or SwissProt), after removal of redundant genes.
(XLS)

Table S3 Table showing lncRNA genes with RPKM >1 in a least one sample. RNA genes >200 nucleotides, annotated in the Human lincRNA catalog, the lncRNA database, and the Noncode database, combined to eliminate redundancies. lncRNA genes up-regulated and down-regulated >2-fold are shaded in green and orange, respectively.
(XLS)

Table S4 Table showing the expressed lncRNAs and their closest expressed protein-coding gene. Expression cutoff was defined as RPKM >1 in at least one sample.
(XLS)

Table S5 Table showing the summary of statistics for the RNA sequencing data.
(DOC)

Table S6 List of publicly available annotations used to generate the coding and noncoding RNA datasets in this study.
(DOC)
