# Supplementary figures and images for: Polyadenylation-Dependent Control of Long Noncoding RNA Expression by the Poly(A)-Binding Protein Nuclear 1
Source: PLoS Genet. 2012 Nov 15;8(11):e1003078. doi: 10.1371/journal.pgen.1003078 (PMC3499365; doi:10.1371/journal.pgen.1003078)

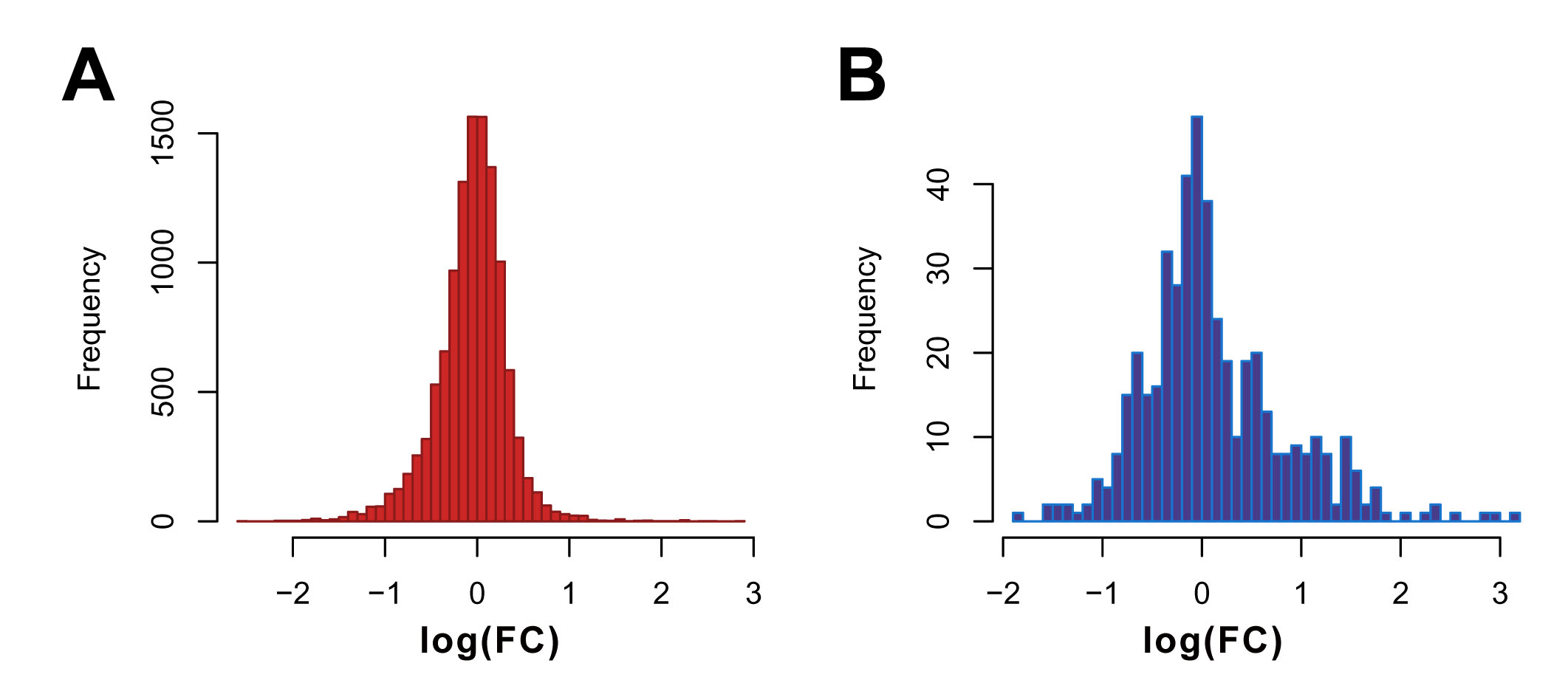


**Figure S1.**

Supplement: Figure S1 — lncRNAs are more affected than mRNAs by a deficiency in PABPN1. Histograms of log ratios/fold-change (FC) in expression levels (as determined using normalized RPKM counts of PABPN1–depleted conditions relative to control siRNA treatment) showing the distribution of (A) the 11,572 protein-coding genes and (B) the 469 lncRNA-coding genes detected RPKM >1 in at least one sample. The density distribution of these histograms was analyzed using Kernel density estimation statistics, yielding the smoothed distribution shown in Figure 2A. (DOC) [file pgen.1003078.s001.doc]

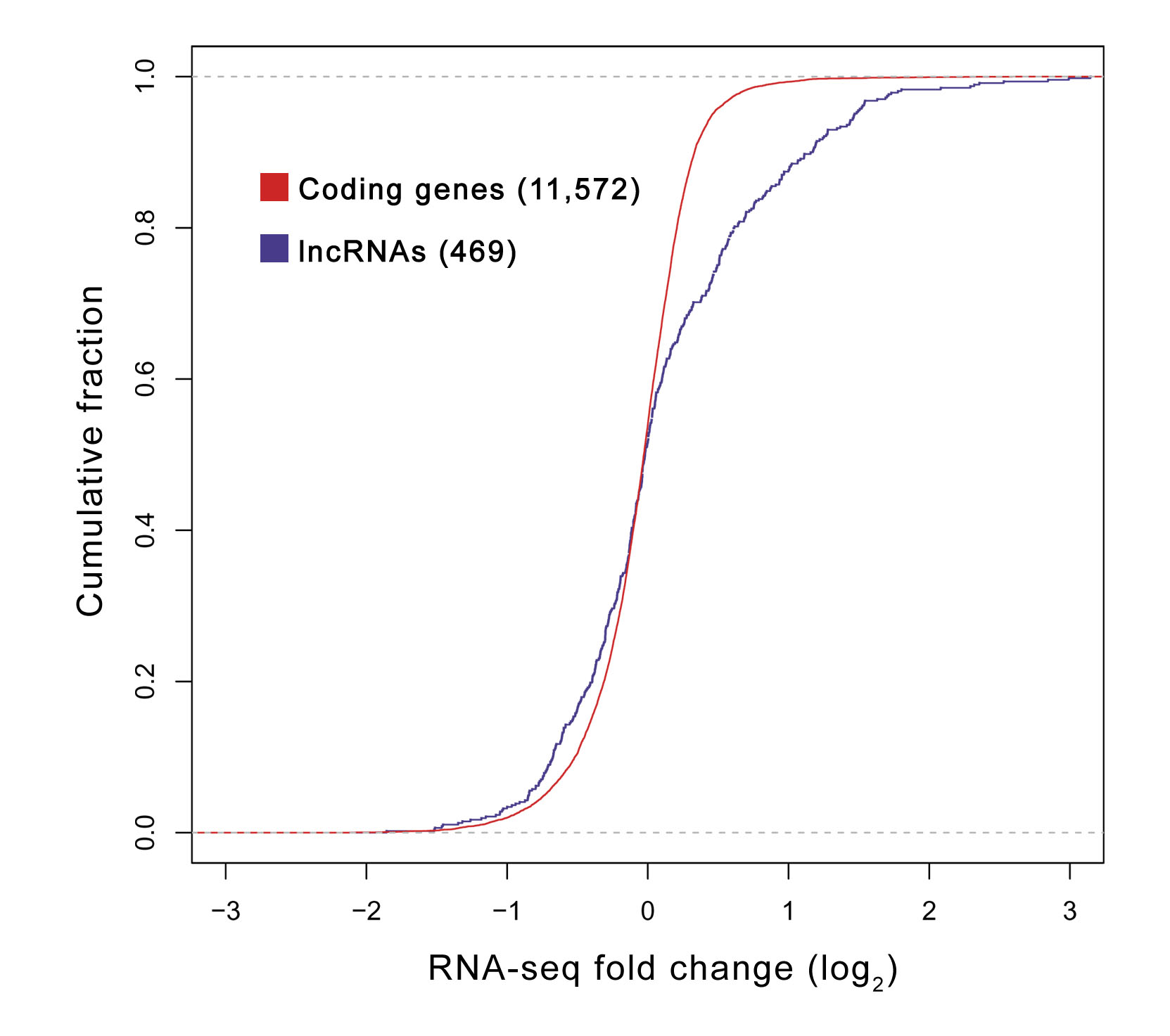
**Figure S2.**

Supplement: Figure S2 — The expression of lncRNA genes is more significantly affected by a deficiency in PABPN1 than protein-coding genes. Cumulative distribution of RPKM fold changes after depleting PABPN1. Plotted are distributions for the protein-coding (red) and lncRNA (blue) genes with RPKM >1. The number of genes in each category is indicated in parentheses. The two distributions are significantly different (Kolmogorov-Smirnov test p-value <2.2 e-16). (DOC) [file pgen.1003078.s002.doc]

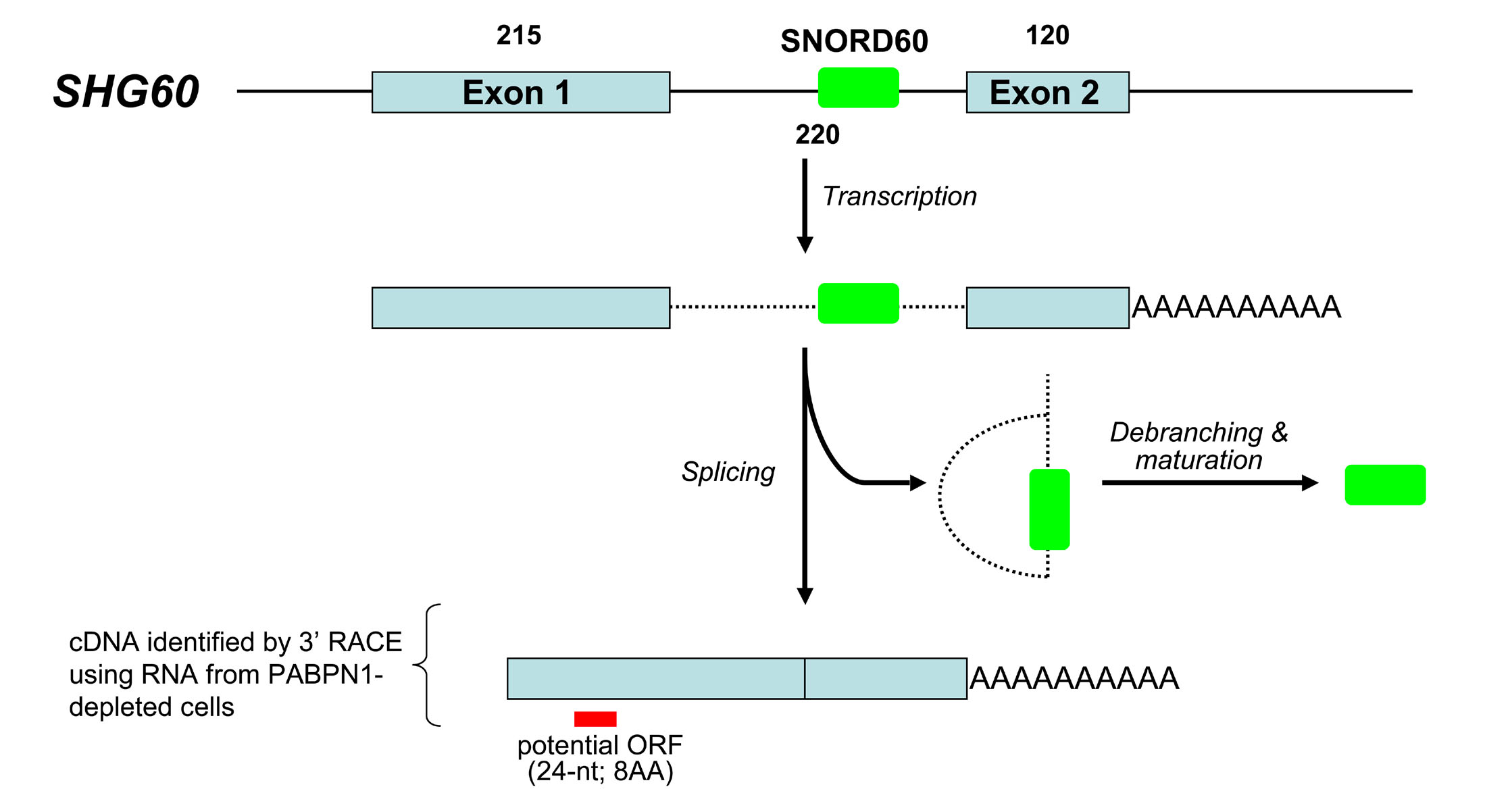
**Figure S3.**

Supplement: Figure S3 — Genomic organization of the SNORD60 Host Gene (SHG60). The blue rectangular boxes represent the 2 exons of human SHG60; the green rectangle represents the SNORD60 snoRNA present in the SHG60 intron. Numbers above and below the gene are the lengths of exons and intron, respectively, as determined by 5′ and 3′ RACE experiments. Transcription of SHG60 produces a 555-nt-long unspliced precursor transcript. Splicing of the SHG60 precursor yields two products: (i) an excised intron that will be processed into the mature C/D box SNORD60 snoRNA, and (ii) a 335-nt-long polyadenylated RNA with limited coding potential (24-nt ORF shown in red). (DOC) [file pgen.1003078.s003.doc]

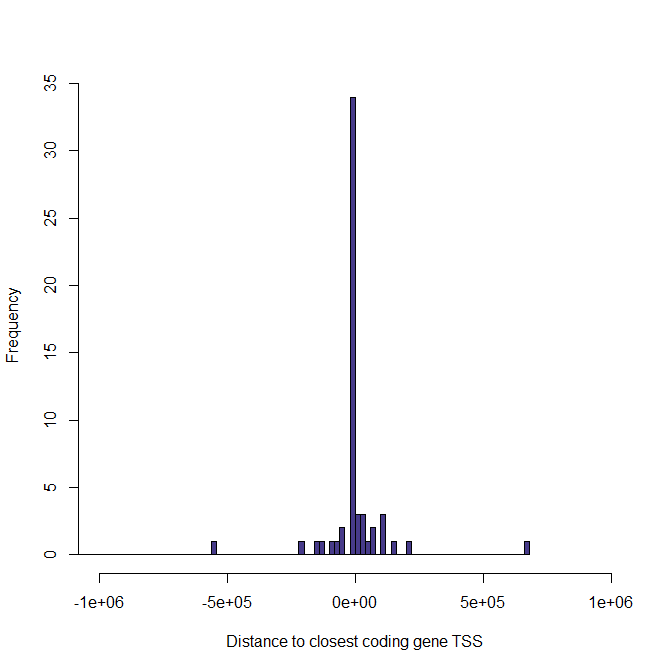
**Figure S4.**

Supplement: Figure S4 — lncRNA genes that are negatively regulated by PABPN1 are frequently located upstream and near the transcription start site of a neighboring protein-coding gene. Histogram showing the distribution of lncRNA genes up-regulated in PABPN1–depleted cells according to the distance from their nearest expressed protein-coding gene. Bin size: 10 Kb. (DOC) [file pgen.1003078.s004.doc]

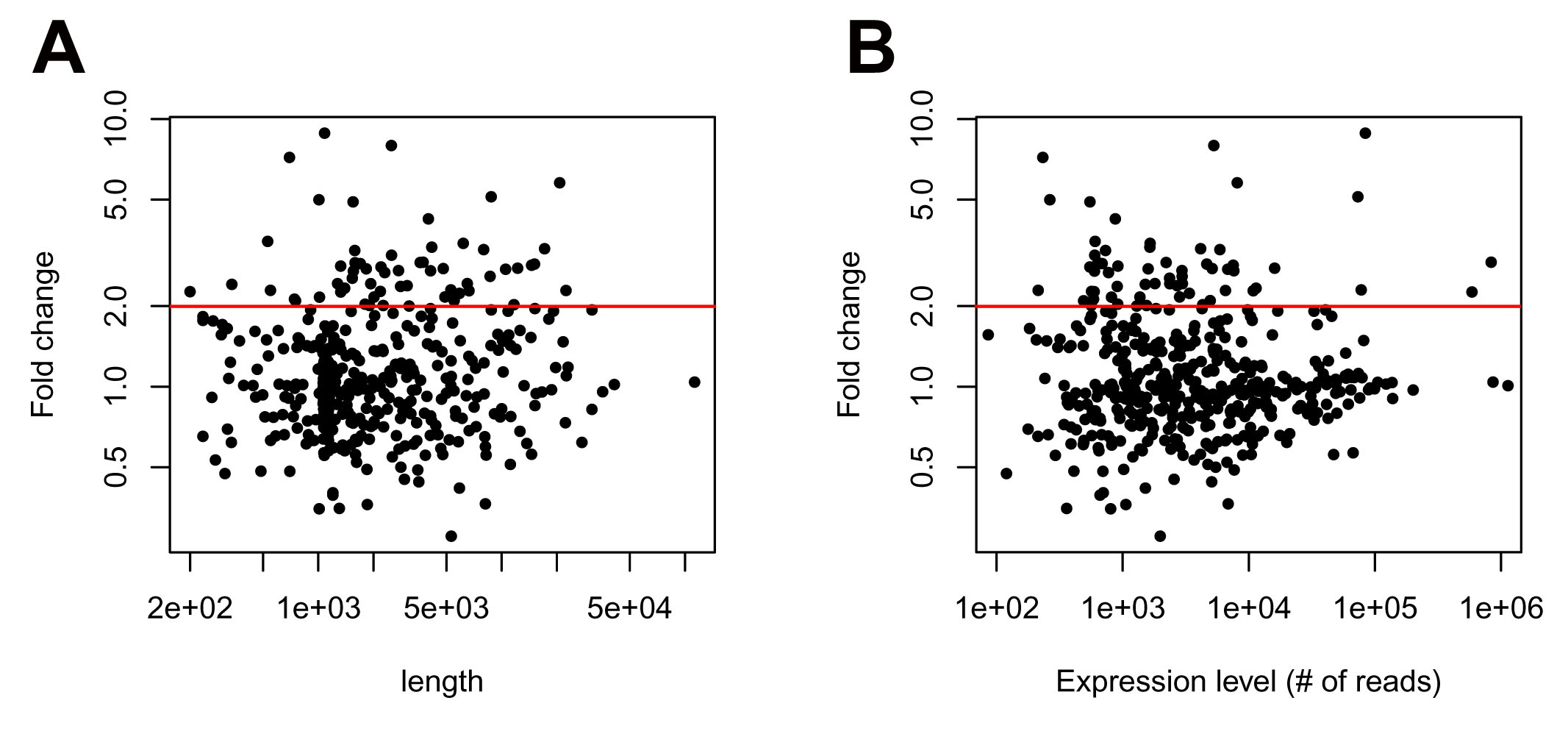


**Figure S5.**

Supplement: Figure S5 — Negative regulation of lncRNA expression by PABPN1 is not correlated with RNA size and expression level. Scatter plots of RNA length (A, in nucleotides, X-axis) or normalized expression levels (B, number of reads, X-axis) versus the fold change in expression in PABPN1–depleted cells relative to control cells (Y-axis), for the 469 lncRNAs expressed >1 RPKM in at least one sample. Dots above the red horizontal line represent the 60 lncRNAs induced >2-fold in PABPN1–deficient cells. (DOC) [file pgen.1003078.s005.doc]

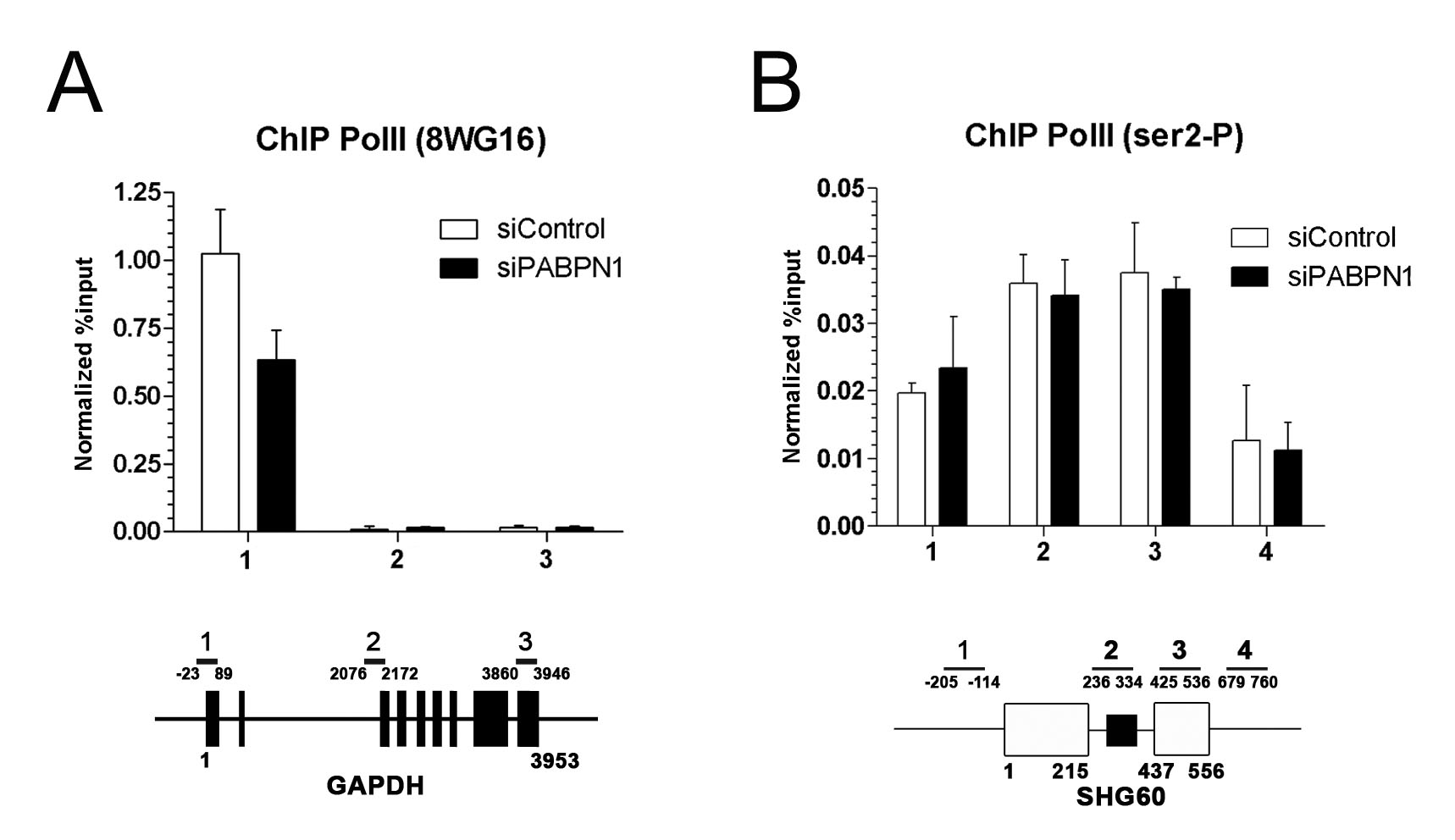


**Figure S6.**

Supplement: Figure S6 — PABPN1 depletion does not affect GAPDH and SHG60 transcription. ChIP assays were performed on extracts prepared from cells treated with control (white bars) and PABPN1–specific (black bars) siRNAs using (A) a monoclonal antibody (8WG16) specific to RNA Pol II and (B) an anti-RNA Pol II antibody specific for Serine-2 phosphorylated CTD repeats. The coprecipitating DNA was quantified by real-time PCR using gene-specific primer pairs located along the GAPDH gene (A) and SHG60 gene (B). ChIP data are presented as percentage of input normalized to control purifications. Values represent the means of at least three independent experiments and bars correspond to standard deviations. (DOC) [file pgen.1003078.s006.doc]

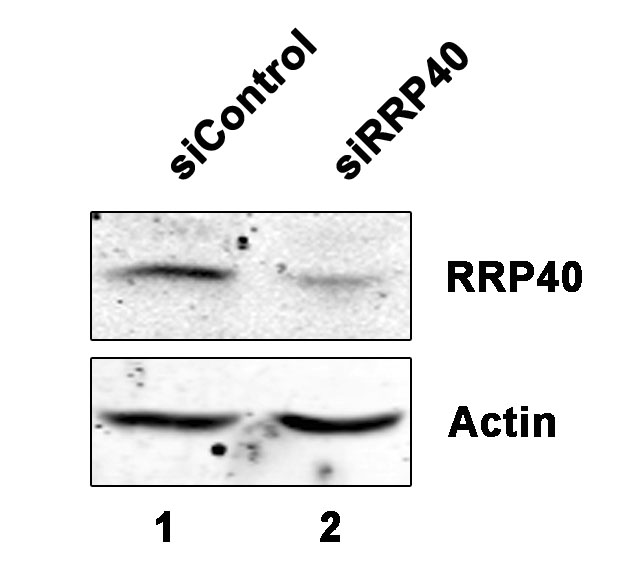
**Figure S7.**

Supplement: Figure S7 — siRNA–mediated depletion of human RRP40. Western blot analysis of total extracts prepared from HeLa cells treated with RRP40-specific (lane 2) and control (lane 1) siRNAs for 72 hrs. (DOC) [file pgen.1003078.s007.doc]

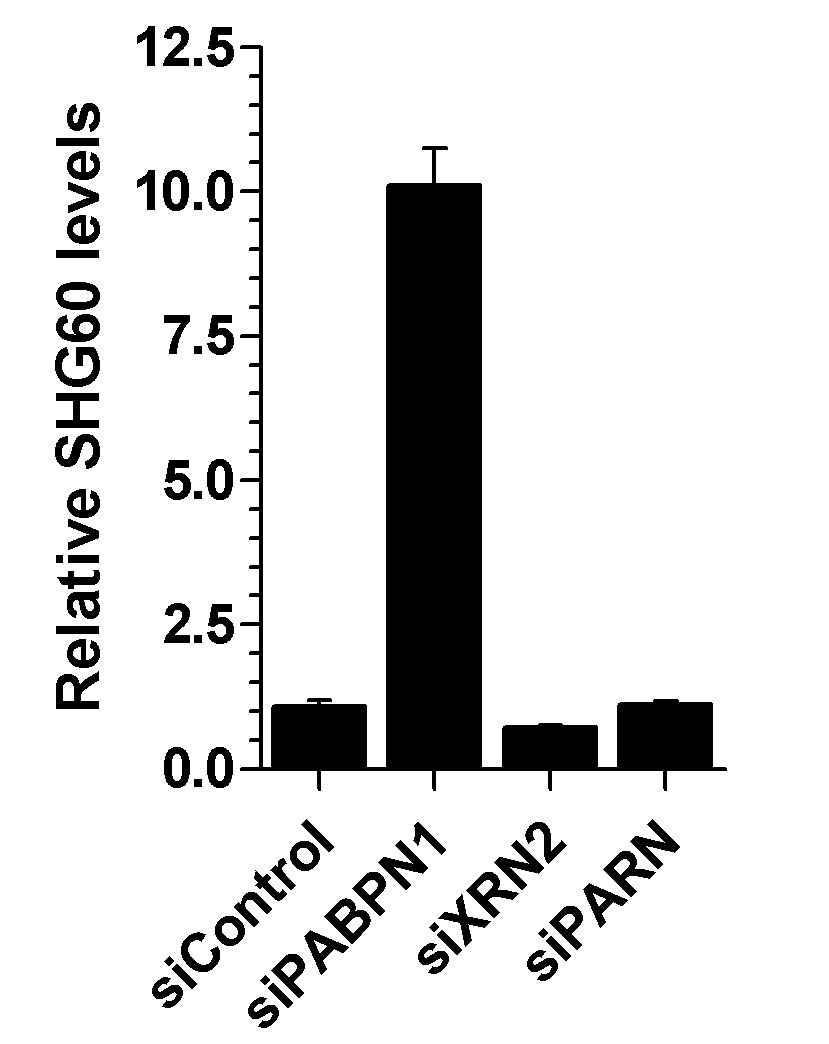


**Figure S8.**

Supplement: Figure S8 — XRN2 and PARN are not required for the control of SHG60 lncRNA expression. Quantitative RT-PCR analysis of RNA prepared from HeLa cells treated with control siRNAs as well as with siRNAs specific to PABPN1, XRN2, and PARN. Fold increases in SHG60 lncRNA levels are relative to control siRNA and normalized to GAPDH mRNA. The data and error bars represent the average and standard deviation from at least three independent experiments. (DOC) [file pgen.1003078.s008.doc]

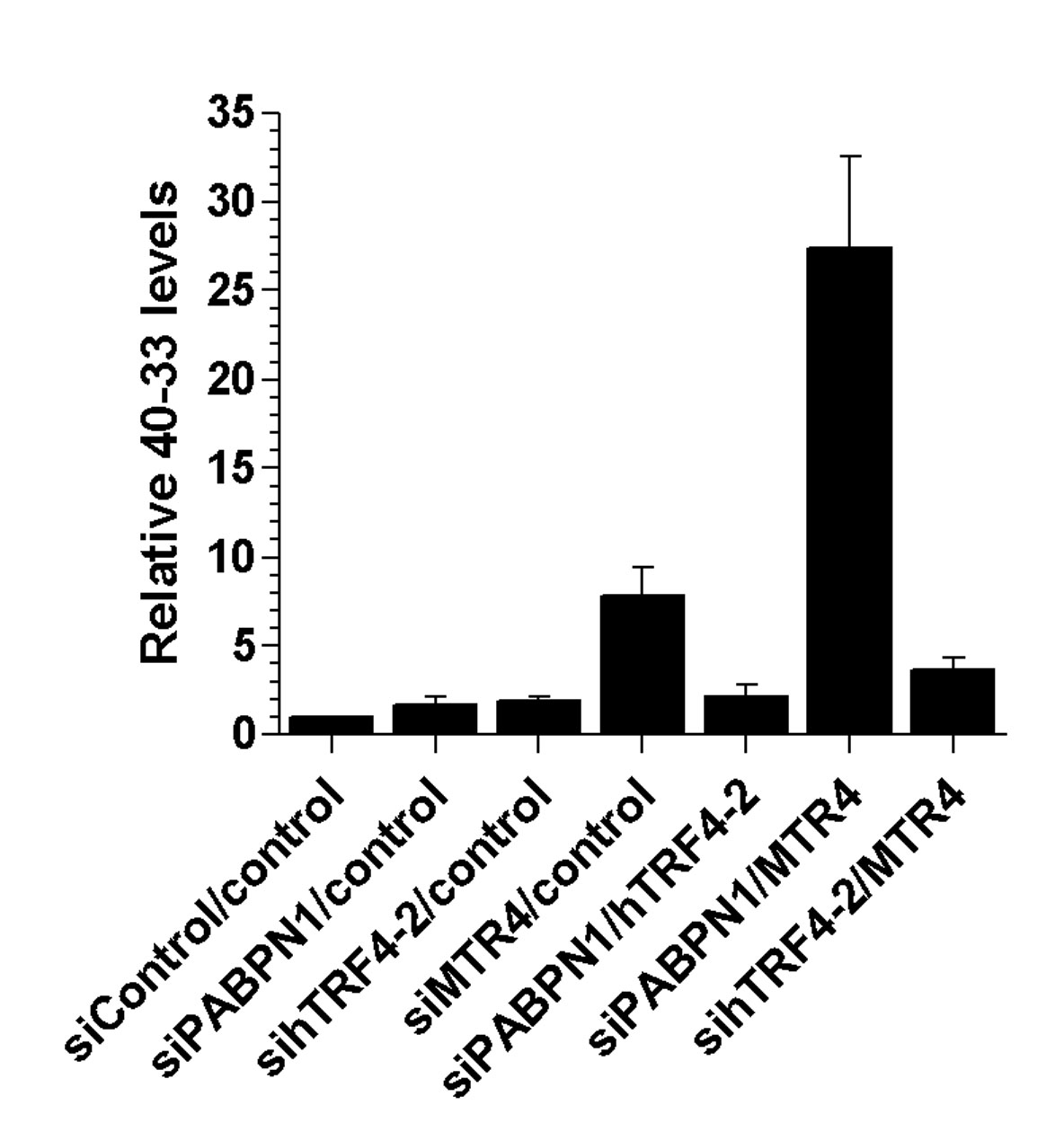


**Figure S9.**

Supplement: Figure S9 — Individual depletion of PABPN1 does not affect the expression of a promoter upstream transcript (PROMPT). Quantitative RT-PCR analysis of a representative PROMPT (40-33) region. HeLa cells were treated with the indicated combinations of siRNAs. Fold increases in 40-33 PROMPT levels are relative to the control/control siRNA mix and normalized to GAPDH mRNA. The data and error bars represent the average and standard deviation from at least three independent experiments. (DOC) [file pgen.1003078.s009.doc]

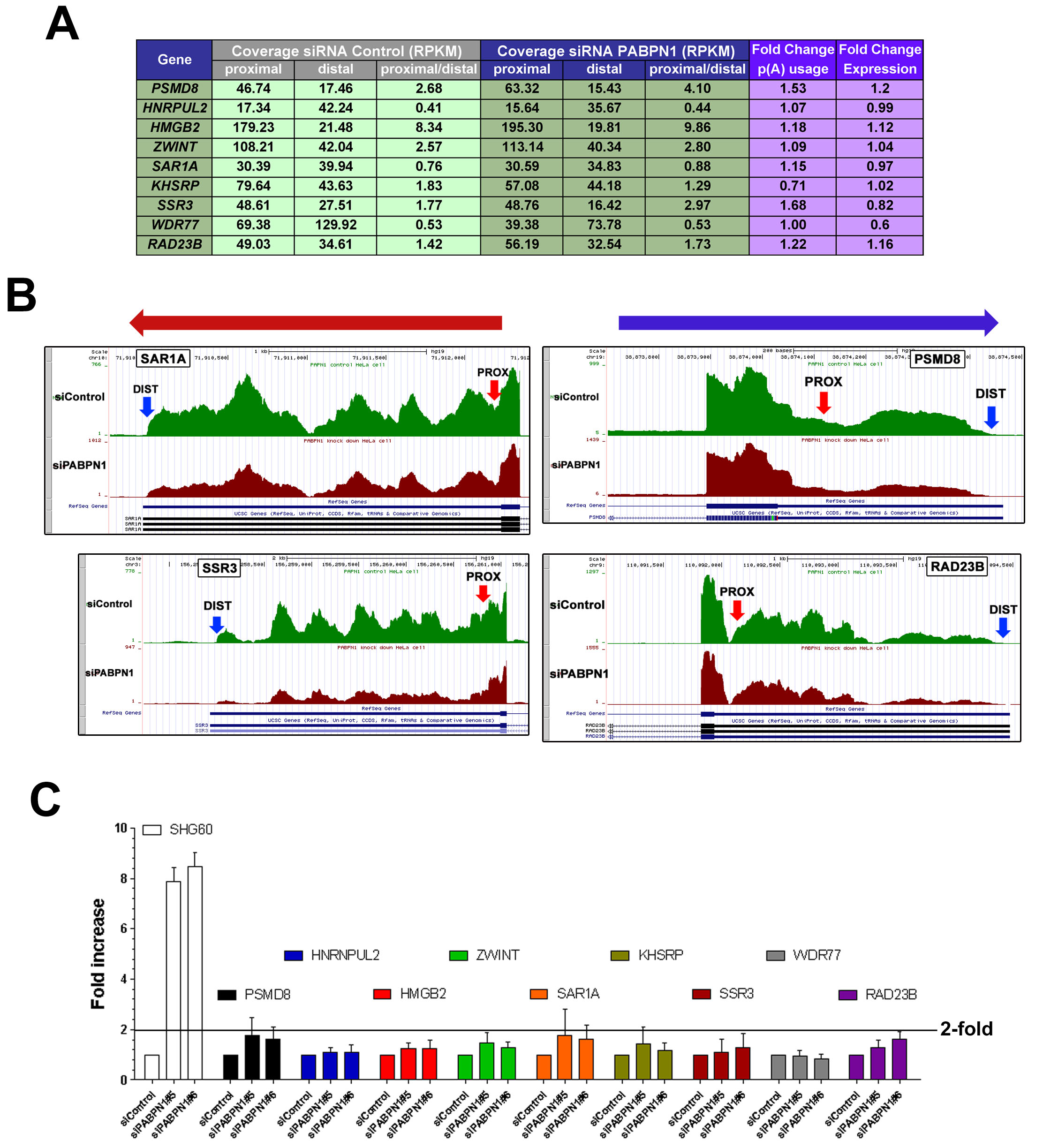


**Figure S10.**

Supplement: Figure S10 — A PABPN1–dependent switch in polyadenylation site usage does not necessarily induce a change in mRNA level. (A) Quantification of the fold changes in proximal/distal poly(A) site usage between PABPN1–depleted and control cells. Proximal/Distal ratios were calculated for the indicated genes using a previously described approach [84] in which 3′ UTRs are partitioned into two segments: a proximal 3′ UTR, delimited by sequences downstream of the STOP codon and upstream of the most proximal poly(A) site, and a distal 3′ UTR, delimited by sequences downstream of the proximal poly(A) site and upstream of the most distal poly(A) site. Coordinates for proximal and distal poly(A) sites were obtained from the poly(A) database [85]. The number of reads mapping to each of these segments was used to calculate RPKM values for proximal and distal 3′ UTRs for PABPN1–depleted and control samples. For eight of the nine tested genes that were previously shown to present a switch in poly(A) site usage upon depletion of PABPN1 [16], we observed increased usage of the proximal polyadenylation site in PABPN1–depleted conditions, in agreement with previous reports. Fold changes in gene expression as determined by RNA-seq are also indicated. (B) Examples of genes showing 3′ UTR shortening, indicated by the reduced level of read coverage downstream of the proximal poly(A) site relative to upstream the proximal poly(A) site in PABPN1–depleted cells compared to control cells. The red and blue arrows above the genes indicate their orientation. (C) Quantitative RT-PCR analysis of RNA prepared from HeLa cells that were treated with control and PABPN1–specific (#5 and #6) siRNAs using sequence-specific primers to the indicated genes. Fold increases are relative to control siRNA-treated cells and normalized to GAPDH mRNA. The SGH60 lncRNA was used as a positive control for PABPN1–dependent upregulation. The data and error bars represent the average and standard deviation from at least three independ [file pgen.1003078.s010.doc]

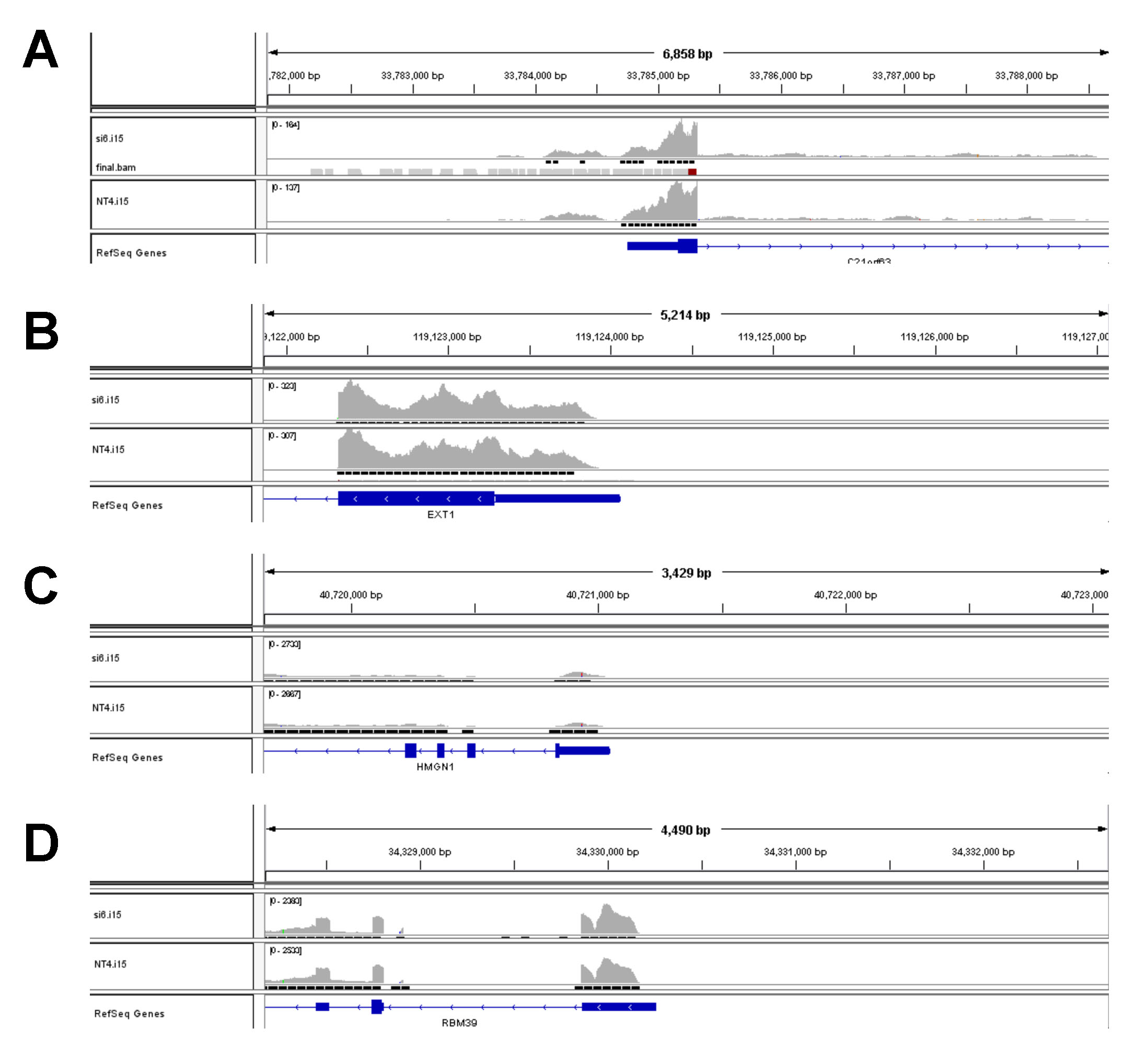


**Figure S11.**

Supplement: Figure S11 — Loss of PABPN1 does not result in the accumulation of promoter upstream transcripts (PROMPTs). (A–D) Integrated Genome Viewer screenshots showing the transcriptional start sites of the C21orf63 (A), EXT1 (B), HMGN1 (C), and RBM39 (D) genes that were previously associated with PROMPT activity [39]. The coverage of the RNA-seq reads (grey) from cells treated with PABPN1–specific (si6; top) and control (NT4; bottom) siRNAs is shown along the RefSeq annotations (blue). (DOC) [file pgen.1003078.s011.doc]
