# Supplementary material for: Polyadenylation-Dependent Control of Long Noncoding RNA Expression by the Poly(A)-Binding Protein Nuclear 1
Source: PLoS Genet. 2012 Nov 15;8(11):e1003078. doi: 10.1371/journal.pgen.1003078 (PMC3499365; doi:10.1371/journal.pgen.1003078)
Supplement: Table S5 — Table showing the summary of statistics for the RNA sequencing data. (DOC) [file pgen.1003078.s016.doc]

**Table S5. Summary of statistics for the RNA sequencing data.**

|  | **siControl** | **siPABPN1#6** |
| --- | --- | --- |
| Total reads | 196,998,210 | 192,880,594 |
| Mapped quality reads | 171,148,013 | 167,589,377 |
| Properly paired | 138,921,672 | 140,875,804 |
| Singletons | 10,277,925 | 10,087,725 |
| Proportion of reads mapped to ncRNA genes (as opposed to protein-coding genes) | 4.7% | 5.5% |
