# Supplementary material for: Polyadenylation-Dependent Control of Long Noncoding RNA Expression by the Poly(A)-Binding Protein Nuclear 1
Source: PLoS Genet. 2012 Nov 15;8(11):e1003078. doi: 10.1371/journal.pgen.1003078 (PMC3499365; doi:10.1371/journal.pgen.1003078)
Supplement: Table S6 — List of publicly available annotations used to generate the coding and noncoding RNA datasets in this study. (DOC) [file pgen.1003078.s017.doc]

**Table S6. List of publicly available annotations used to generate the coding and noncoding** RNA datasets.

| **Dataset name** | **Downloaded from (date)** | **References** | **Number of records** | **Note** |
| --- | --- | --- | --- | --- |
| UCSC genes | The UCSC genome browser,  built NCBI137/hg19 (Dec. 2010) | Rhead B, et al. (2010) Nucleic Acids Res 38: D613-619. | 77614 |  |
| lincRNAs | Human lincRNA Catalog, Broad Institute http://www.broadinstitute.org/genome_bio/human_lincrnas (Sept. 2011) | Cabili MN, et al. (2011) Genes Dev 25: 1915-1927. | 14353 |  |
| lncRNAs | The long non-coding RNA database, http://lncrnadb.com (Jul. 2011) | Amaral PP, et al. (2011) Nucleic Acids Res 39: D146-151. | 74 |  |
| noncode | Noncode Database, v3.0, http://www.noncode.org (Jul. 2011) | He S, et al. (2008) Nucleic Acids Res 36: D170-172. | 857 | piRNAs were removed from the analysis. |
| snoRNAs | Functional RNA Database,  http://www.ncrna.org/frnadb (Sept. 2011) | Mituyama T, et al. (2009) Nucleic Acids Res 37: D89-92. | 402 | Annotated regions were extended by 500-bp  upstream and downstream of the annotation, to include the flanking exons of the genes containing the snoRNAs. 1000-bp, 1500-bp, 3000-bp, and 5000-bp were also tested with similar results. |
